# Characterisation of complexes formed by parasite proteins exported into the host cell compartment of *Plasmodium falciparum* infected red blood cells

**DOI:** 10.1111/cmi.13332

**Published:** 2021-05-03

**Authors:** Thorey K. Jonsdottir, Natalie A. Counihan, Joyanta K. Modak, Betty Kouskousis, Paul R. Sanders, Mikha Gabriela, Hayley E. Bullen, Brendan S. Crabb, Tania F. de Koning‐Ward, Paul R. Gilson

**Affiliations:** ^1^ Burnet Institute Melbourne Australia; ^2^ Department of Microbiology and Immunology University of Melbourne Melbourne Australia; ^3^ School of Medicine Deakin University Waurn Ponds Australia; ^4^ Monash Micro‐imaging Monash University Melbourne Australia; ^5^ Department of Microbiology Monash University Melbourne Australia

**Keywords:** exported proteins, J‐dots, malaria, Maurer's clefts, new permeability pathways, *Plasmodium falciparum*, RhopH

## Abstract

During its intraerythrocytic life cycle, the human malaria parasite *Plasmodium falciparum* supplements its nutritional requirements by scavenging substrates from the plasma through the new permeability pathways (NPPs) installed in the red blood cell (RBC) membrane. Parasite proteins of the RhopH complex: CLAG3, RhopH2, RhopH3, have been implicated in NPP activity. Here, we studied 13 exported proteins previously hypothesised to interact with RhopH2, to study their potential contribution to the function of NPPs. NPP activity assays revealed that the 13 proteins do not appear to be individually important for NPP function, as conditional knockdown of these proteins had no effect on sorbitol uptake. Intriguingly, reciprocal immunoprecipitation assays showed that five of the 13 proteins interact with all members of the RhopH complex, with PF3D7_1401200 showing the strongest association. Mass spectrometry‐based proteomics further identified new protein complexes; a cytoskeletal complex and a Maurer's clefts/J‐dot complex, which overall helps clarify protein–protein interactions within the infected RBC (iRBC) and is suggestive of the potential trafficking route of the RhopH complex itself to the RBC membrane.

1

**Take Away**

- New protein complexes were identified that provide a clearer picture of the *Plasmodium falciparum exported protein network*.
- Five proteins were confirmed to interact with RhopH components: RESA1, PF3D7_1201000, PF3D7_0401800, LyMP and PF3D7_1401200.
- The export and localisation of six new proteins previously hypothesised to be exported into the red blood cell cytoplasm were validated by immunofluorescence analysis.

## 2 INTRODUCTION

Malaria is a febrile illness caused by *Plasmodium* parasites. It remains a major global health problem and was estimated to cause 229 million cases in 2019, leading to the death of approximately half a million people, most of whom were children under the age of five (W.H.O, 2020). *P. falciparum*, the causative agent of the most severe form of malaria in humans, is renowned for its ability to manipulate and modify the red blood cells (RBCs) of its human host for its survival by exporting hundreds of parasite proteins into the RBC cytosol [reviewed in (de Koning‐Ward et al., 2016)]. The infected RBC (iRBC) becomes more rigid and adherent to the microvascular endothelium, preventing its circulation through the spleen and subsequent immune destruction [reviewed in (Tilley et al., 2011)]. Parasite structures are formed in the iRBC to traffic parasite proteins within the host cell. These include Maurer's clefts (MCs) which are membranous structures involved in protein cargo sorting, and J‐dots, which are mobile structures affiliated with heat shock proteins and trafficking of the major virulence protein PfEMP1 (Behl et al., 2019; Kulzer et al., 2010, 2012; Lanzer et al., 2006). The iRBC also becomes more permeable to plasma nutrients including isoleucine and pantothenic acid to supplement rapid parasite growth (Boddey & Cowman, 2013, Cooke et al., 2004, Gilson et al., 2017, Kirk et al., 1994, Kirk & Horner, 1995).

The establishment of nutrient channels in the iRBC membrane, collectively referred to as the new permeability pathways (NPPs), renders the iRBC permeable to various solutes (Ginsburg et al., 1985, Staines et al., 2000, Upston & Gero, 1995). These influx/efflux channels allow the parasite to gain access to essential extracellular plasma nutrients [reviewed in (Saliba & Kirk, 2001)], and aid in maintaining optimum physiological stability of the infected host cell (Lew et al., 2003).

Through conditional drug resistance, knockdown studies and nutrient uptake assays, NPP activity has been affiliated with the functioning of three rhoptry proteins, those being RhopH1 (CLAG3) (Ito et al., 2017; Nguitragool et al., 2011), RhopH2 (Counihan et al., 2017; Ito et al., 2017) and RhopH3 (Sherling et al., 2017). RhopH1 is encoded by a multigene family comprising *clag2*, *clag3.1*, *clag3.2*, *clag8* and *clag9*, whilst RhopH2 and RhopH3 are encoded by single genes (Kaneko et al., 2001, 2005). The RhopH1 genes *clag3.1* and *clag3.2* are mutually exclusively transcribed and are the only *clags* affiliated with NPP activity so far, but are not essential for parasite survival (Comeaux et al., 2011; Nguitragool et al., 2011). RhopH2 and RhopH3 are both refractory to genetic disruption (Cowman et al., 2000; Ito et al., 2017) and protein knockdown results in severe growth reduction through decreased nutrient uptake and in the case of RhopH3 knockdown also results in invasion defects (Counihan et al., 2017; Ito et al., 2017; Sherling et al., 2017).

It remains unclear if the RhopH complex alone is sufficient to carry out NPP functions or if other proteins are additionally required (Ito et al., 2017). NPP function has also been shown to depend on the *Plasmodium* translocon of exported proteins (PTEX) (Beck et al., 2014), which resides in the parasitophorous vacuole (PV) and exports proteins across the PV membrane (PVM) and into the host cell (de Koning‐Ward et al., 2009). There is conflicting evidence whether the RhopH complex itself requires PTEX for export into the RBC or if other exported proteins are needed for correct NPP functioning (Ahmad et al., 2020; Beck et al., 2014; Ito et al., 2017). It is also unclear how the RhopH complex is delivered from the PV/PVM to the iRBC periphery, but studies have shown that RhopH2 and RhopH3 are needed for the correct trafficking of CLAG3 to the surface (Ahmad et al., 2020, Ito et al., 2017).

A recent study has revealed that RhopH2 co‐immunoprecipitates 30 proteins predicted to be exported via PTEX, raising the question of whether these proteins could possess NPP functions as well (Counihan et al., 2017). These exported proteins include *Plasmodium* helical interspersed subtelomeric (PHIST) proteins (Sargeant et al., 2006), MC proteins (Lanzer et al., 2006) and cytoskeletal components. Here, we studied 13 of the predicted RhopH2‐interacting exported proteins to confirm their potential role in NPP function and affiliation with RhopH components. Sorbitol‐based NPP assays together with conditional protein knockdown assays revealed that none of the 13 proteins appear to play a major role in NPP functioning. However, reciprocal immunoprecipitation assays show that five of the selected exported proteins co‐precipitate RhopH components, four of which also interact with cytoskeletal components. Furthermore, we have expanded the repertoire of known exported proteins to include; PTP4 (PF3D7_0730900), PF3D7_0532300, PF3D7_1477500, Hyp1 (PF3D7_0301600), PF3D7_0113200, PF3D7_0501000 and PF3D7_1401200. Lastly, mass spectrometry‐based proteomics analysis for 10 of the exported proteins reveals new networking clusters of exported proteins within the iRBC and identifies potential novel complexes involved in cytoskeletal activity, as well as a transient trafficking hub connecting J‐dots and MCs structures. Given the association of RhopH proteins with both MC and J‐dot residential proteins, we propose that these structures are involved in the trafficking of the RhopH complex to the RBC membrane where the NPPs are established.

## 3 RESULTS

### 3.1 Generation of transgenic parasite lines

Here, we focus on 13 of the 30 exported proteins found to co‐precipitate with RhopH2 (Counihan et al., 2017) (Table S1, grey). Proteins were chosen based on RhopH2 association and the ease of generating the gene constructs. 3D7 transgenic parasite lines were generated using either the selection‐linked integration (SLI) method (Birnbaum et al., 2017) or via standard homologous recombination techniques (Crabb et al., 1997) (Figure S1a). Diagnostic PCR was used to confirm that the 13 proteins had been correctly tagged with both a C‐terminal HA‐epitope tag (for protein detection), and a *glmS* ribozyme (for conditional protein knockdown) (Figure S1b,c). Subsequent western blots of tagged lines probed with anti‐HA revealed that all 13 parasite lines displayed a HA‐epitope band of the expected size indicating successful targeting of the genes of interest (Figure S1d). For the SLI constructs, a band ~30 kDa larger than expected for the protein of interest was often observed, which is likely a fusion of the exported protein and neomycin resistance protein formed as a result of incomplete ribosome skipping between the two coding sequences that are separated by a 2A sequence (Figure S1d).

### 3.2 Localisation of the 13 exported proteins within the host cell

**FIGURE 1:**
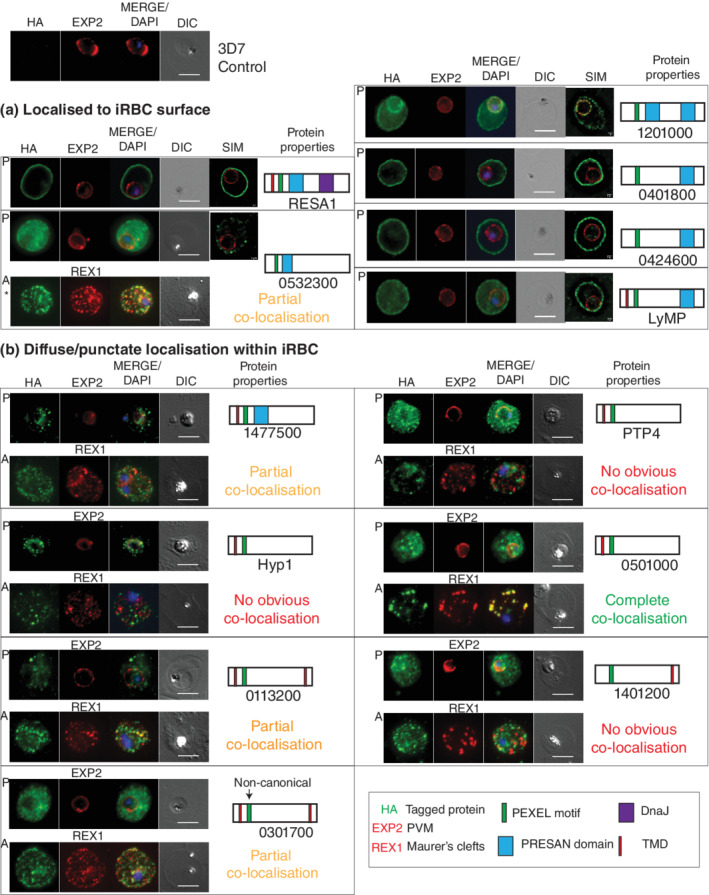
Localisation of the 13 exported proteins. Localisation of the 13‐tagged proteins was detected via indirect immunofluorescence assays of the trophozoite stage iRBCs, when the NPP activity is greatest. All of the HA‐tagged proteins were exported into the iRBC, where (a) five proteins: RESA1, PF3D7_0532300, PF3D7_1201000, PF3D7_0401800 and LyMP showed surface localisation. Structured Illumination Microscopy (SIM) analysis was used to obtain higher spatial resolution. PF3D7_0532300 (*) displayed either clear surface localisation, or appeared as discrete puncta at the surface and diffused throughout the iRBC cytoplasm depending on the fixation used. (b) Seven proteins showed diffuse iRBC signal resembling Maurer's clefts and showed either no, partial or complete co‐localisation with the MCs marker REX1. Scale bars = 5 μm or 1 μm (SIM). Predicted domain structures of each protein are indicated to the right of their microscopy image found on PlasmoDB. 3D7 (top left) was used as negative control for HA signal (tagged protein) and EXP2 was used as PVM marker throughout. P stands for paraformaldehyde/glutaraldehyde fixation and A for acetone/methanol fixation

Indirect immunofluorescence assays were used to confirm the localisation of the 13 proteins (Figure 1). RESA1, PF3D7_1201000, PF3D7_0401800, PF3D7_0424600 and LyMP displayed a strong signal at the surface of the iRBC, which was often accompanied by a weaker signal in the cytoplasm (Figure 1a), in agreement with previous studies (Davies et al., 2016; Moreira et al., 2016; Proellocks et al., 2014; Tarr et al., 2014; Tiburcio et al., 2015). PF3D7_0532300 has been previously suggested to localise to the MCs (Moreira et al., 2016), which we confirm here through co‐localisation with the MC marker REX1 but we also observed signal lining the iRBC surface (Figure 1a). The remainder of the proteins analysed displayed a diffuse or punctate signal throughout the iRBC, with PF3D7_0501000 showing complete co‐localisation with REX1 whilst the others showed only partial or no co‐localisation with REX1 (Figure 1b). Some proteins also showed partial co‐localisation with the J‐dot resident protein PF3D7_0801000 (Zhang et al., 2017) (Figure S2). Super‐resolution microscopy was used to gain better resolution of surface protein localisation. RESA, PF3D7_0401800, PF3D7_0424600 and LyMP showed a more continuous signal around the iRBC surface, whereas PF3D7_0532300 and PF3D7_1201000 showed a punctate signal around the surface, indicating they might be restricted to specific zones (Figure 1a).

### 3.3 Partial knockdown of the 13 exported proteins did not affect NPP activity

**FIGURE 2:**
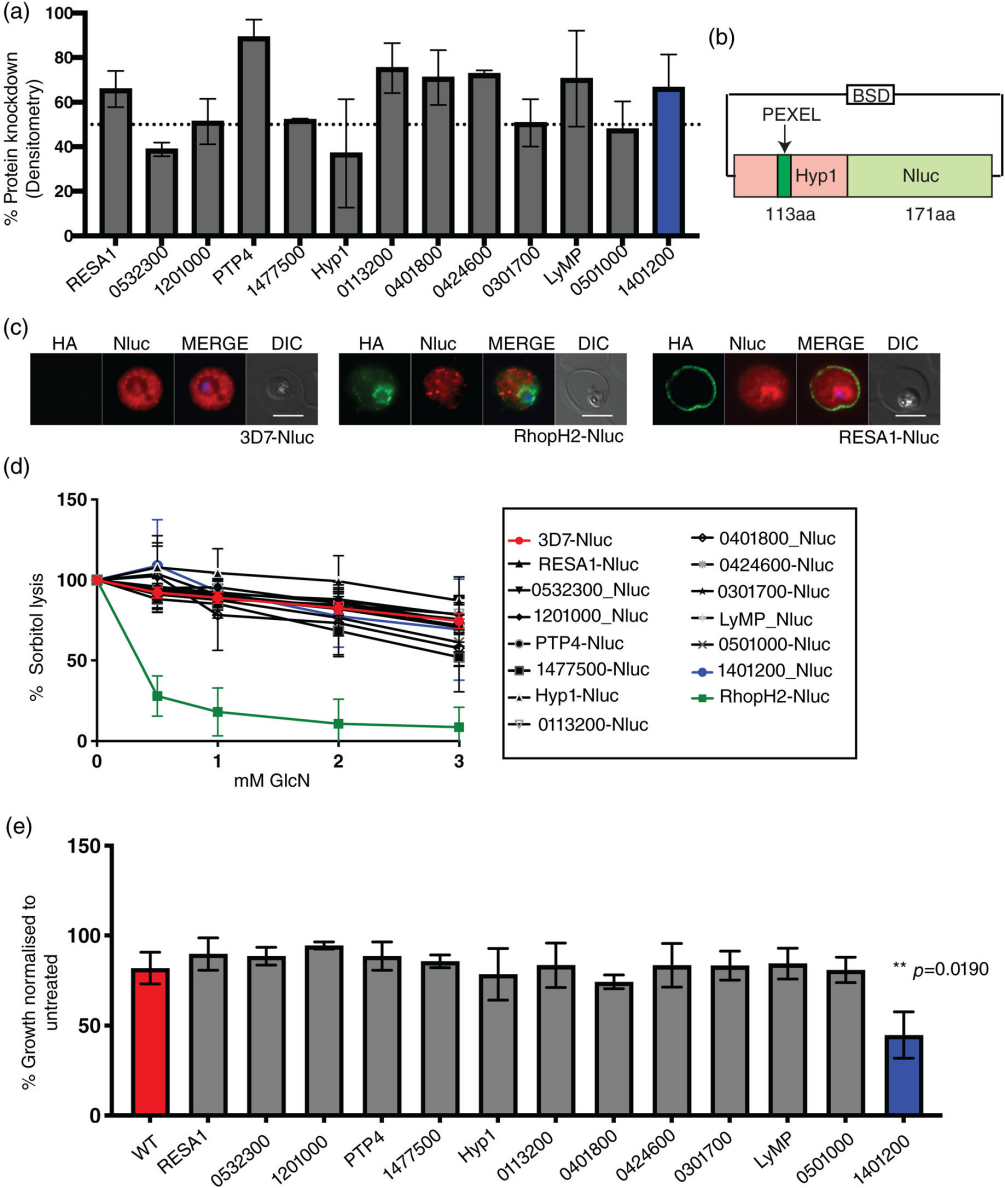
Conditional knockdown of the 13 proteins indicate none of the proteins are important for NPP function and only PF3D7_1401200 shows growth phenotype. (a) Western blots were performed to assess level of protein knockdown when *glmS*‐tagged trophozoite stage parasites were treated with 2.5 mM glucosamine (GlcN) over one cell cycle. Densitometry was used to measure the level of protein knockdown (shown on graph), where individual blots can be found in Figure S3a. Error bars represent SD for 2 biological replicates. (b) Schematic of the exported Nluc reporter construct transfected into the 13 transgenic parasite lines. Nluc is N‐terminally appended with the PEXEL encoding region of exported protein Hyp1 (PF3D7_0113300) and is thus exported into the iRBC. (c) The 13 transgenic parasite lines containing exported Nluc reporter were harvested at trophozoite stage when the exported Nluc was expressed and indirect immunofluorescence assays were used to confirm that the Nluc was successfully exported into the iRBC. HA represents the target protein and Nluc represents the exported Nluc. 3D7 and RhopH2‐HAglmS parasites containing exported Nluc were used as controls. Scale bars = 5 μm. Only representative figures are shown, complete data set can be found in Figure S3b. (d) Trophozoite stage parasites were treated with different concentrations of GlcN. After one cycle parasites were harvested and NPP activity was assessed via sorbitol lysis assays. The percentage of sorbitol lysis measured in relative light units (RLU) released per minute of lysis is shown on the *y*‐axis. 100% lysis was set as RLU/min in parasites not treated with GlcN. 3D7 wild‐type parasites (red) with an exported Nluc were used as negative control for sorbitol lysis and RhopH2‐Nluc (green) are an NPP knockdown control. This data was overlaid on the RhopH2‐HA binding proteins data indicated in black. None of the 13 parasite lines were found to have decreased sorbitol lysis after GlcN treatment when compared to wild‐type parasites. The experiment was repeated on three independent occasions, using three technical replicates and error bars represent SD. (e) Trophozoite stage parasites were treated with different concentrations of GlcN over three consecutive parasite cycles. PF3D7_1401200 showed 50% reduction in parasite growth in the third cycle of treatment when compared to the 3D7 control. Data represent three biological replicates completed in three technical replicates. Error bars represent SD. Statistical analysis was performed using an unpaired *t* test (Welch's *t* test). The graph only shows third cycle of treatment, 2.5 mM GlcN. All cycles for individual proteins can be found in Figure S4

To perform functional studies with the 13 proteins, parasites were treated with glucosamine (GlcN), to activate the *glmS* ribozyme, resulting in reduced protein expression (Prommana et al., 2013). Trophozoite iRBCs were treated for one cell cycle ±2.5 mM GlcN and subsequent western blots of the GlcN‐treated parasites indicated protein expression was reduced by approximately 40–80% depending on protein, as measured via densitometry (Figures 2a and S3a).

To assess NPP activity, sorbitol lysis sensitivity was measured (Counihan et al., 2017; Nguitragool et al., 2011; Wagner et al., 2003). Specifically, we transfected the 13 parasite lines with an exported nanoluciferase (Nluc), which can be utilised as a read‐out for the percentage parasite lysis following treatment with sorbitol (Figure 2b) (Azevedo et al., 2014; Counihan et al., 2017). Immunofluorescence assays confirmed the presence of exported Nluc in all 13 lines (Figures 2c and S3b).

Trophozoite iRBC was treated with increasing concentrations of GlcN for one cycle to knockdown expression of each tagged protein, prior to incubation in isotonic sorbitol lysis buffer containing NanoGlo, the substrate of Nluc. Sorbitol‐mediated lysis of iRBCs results in release of the exported Nluc which emits bioluminescence in the presence of NanoGlo and can be used as a direct indicator of NPP activity. We observed no changes in sorbitol lysis sensitivity in relative light units (RLU)/min at any GlcN concentration for the 13 proteins studied when compared to wild‐type parasites (Figure 2d, in red). In contrast, RhopH2‐HA*glmS* parasites expressing the exported Nluc reporter, exhibited a strong decrease in bioluminescence signal relative to wild‐type parasites consistent with its involvement in NPP activity (Counihan et al., 2017) (Figure 2d, in green).

We next assessed if this level of protein knockdown (40–80%) was sufficient to reduce parasite proliferation. Trophozoite‐stage parasites were treated with different concentrations of GlcN and harvested each cycle at trophozoites stage over three consecutive cell cycles (7 days). Lactate dehydrogenase (LDH) assays were then performed to measure parasite growth as previously described (Makler & Hinrichs, 1993; Persson et al., 2006) and compared to 3D7 parasites (Figures 2e and S4). Although growth was reduced in the presence of 2.5 mM GlcN over time, only PF3D7_1401200 exhibited significant growth defect by the end of the third cycle of treatment (Figure 2e, individual graphs for each cycle can be found in Figure S4).

### 3.4 Immunoprecipitation assays indicate that five proteins co‐precipitate RhopH components

**FIGURE 3:**
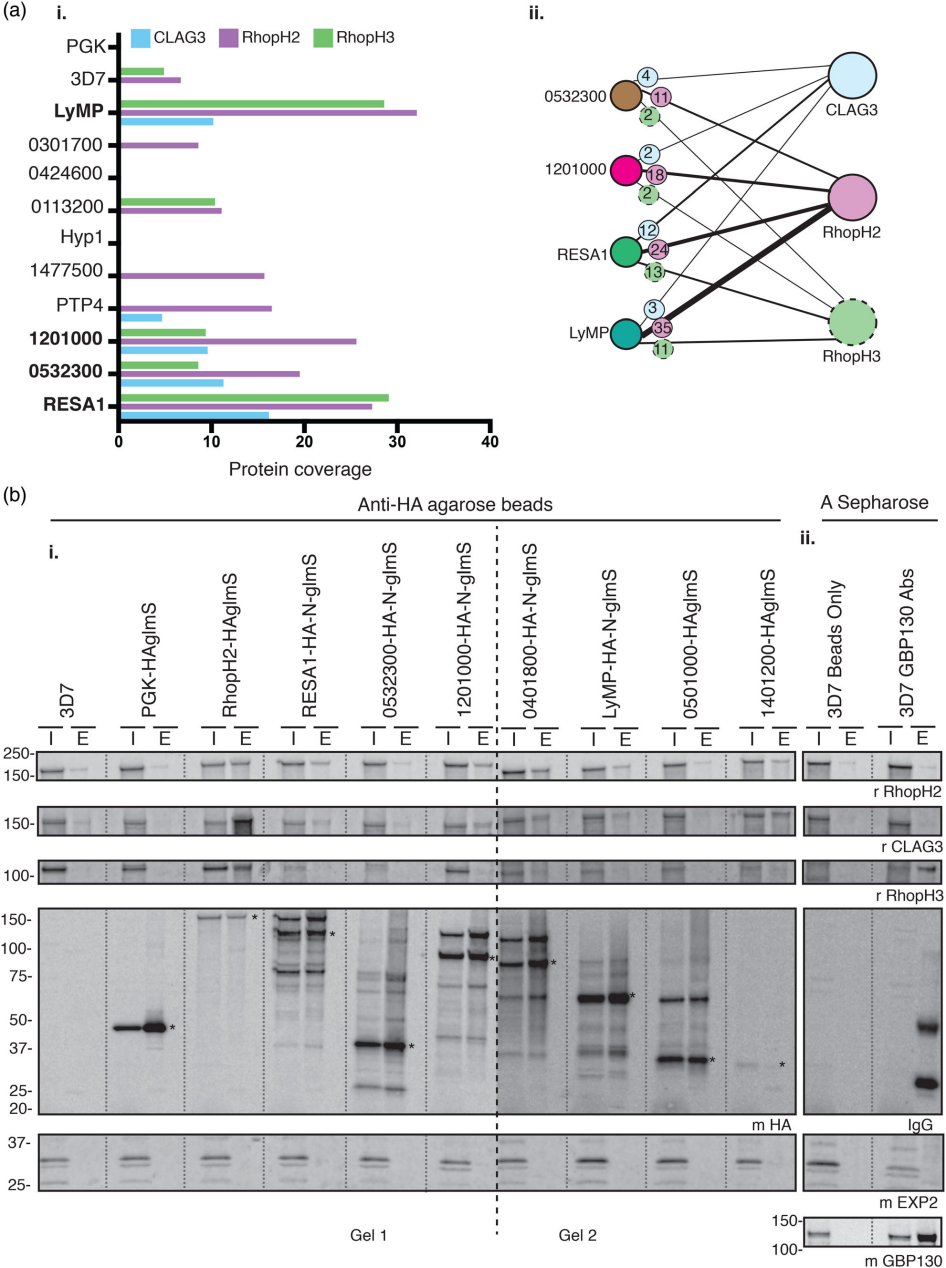
Reciprocal immunoprecipitations indicate five proteins out of 13 co‐precipitate RhopH2. (a) Mass spectrometry analysis of the 10 proteins targeted for reciprocal immunoprecipitations revealed that only four proteins co‐immunoprecipitated all RhopH components, when considering (i) protein coverage (% coverage shown on the *x*‐axis) and (ii) peptide counts. Peptide numbers are shown inside coloured circles, where blue represents CLAG3 peptides, purple RhopH2 peptides and green RhopH3 peptides co‐precipitated by the four proteins targeted in the assay. Thickness of the bands represents protein association, where higher peptide counts have thicker bands. RESA1 and LyMP showed the strongest association with RhopH components whilst PF3D7_0532300 and PF3D7_1201000 showed intermediate interaction. (b) (i) Western blot analysis of reciprocal immunoprecipitations was used to confirm the mass spectrometry data, where RhopH2‐HA*glmS* was used as positive control and 3D7 and PGK‐HA*glmS* as negative controls. RhopH2, RESA1, PF3D7_1201000, PF3D7_0401800, LyMP and PF3D7_1401200 were able to co‐precipitate RhopH2 in more quantity compared to negative control lines. * represents the target protein size. (ii) GBP130 was also targeted for immunoprecipitation using IgG antibody to target native GBP130, where 3D7 incubated with beads only was used as a control. GBP130 only co‐precipitated RhopH3. I; sample input, E; elution

The interaction between the 13 proteins and the RhopH complex was validated using reciprocal immunoprecipitation. RBCs infected with trophozoite stage parasites were isolated on magnetic columns and pellets lysed in 0.25% Triton X‐100. Lysates were incubated with anti‐HA IgG agarose beads and the bound proteins eluted. As a control, wild‐type 3D7 parasites and transgenic parasites with an irrelevant HA‐tagged phosphoglycerate kinase protein (PGK‐HA) were used to detect non‐specific binding to the anti‐HA beads. Interactions were detected by mass spectrometry‐based proteomics for 10 proteins (excluding PF3D7_0401800, PF3D7_0501000 and PF3D7_1401200). Peptide counts were used to semi‐quantitatively determine the degree of protein–protein interaction as well as protein coverage (Figure 3a, Tables 1 and S2).

**TABLE 1:** Mass spectrometry analysis of reciprocal immunoprecipitation assays

(a)									
Interacting Proteins ↓	Proteins Targeted for Immunoprecipitation								
	RhopH2	CLAG3	3D7‐1	RESA1	0532300	1201000	LyMP	3D7‐2	PGK
CLAG3	203	319	12	15	4	2	3	1	0
RhopH2	48	47	9	24	11	18	35	1	0
RhopH3	102	55	12	13	2	2	11	1	0
RESA1*	8	58	7	92	12	13	0	0	0
0532300*	3	0	0	11	26	6	0	2	1
1201000*	3	2	0	20	5	44	0	0	1
LyMP*	31	3	2	12	12	10	58	5	1
0401800	2	11	4	10	8	5	2	1	0
1401200	10	1	0	1	6	1	0	0	0

(b)							
Interacting Proteins ↓	Proteins Targeted for Immunoprecipitation						
	RhopH2	CLAG3	3D7‐1	RESA1	1201000	3D7‐2	PGK
RESA1#	8	58	7	92	13	0	0
1201000#	3	2	0	20	44	0	1
MESA#	46	9	3	35	36	8	1
0936800#	6	0	0	26	39	0	0
Ankyrin	173	220	78	163	134	7	0
Spectrin	116	512	100	377	600	11	10
Band 3	49	90	15	50	33	2	0

(c)						
Interacting Proteins ↓	Proteins Targeted for Immunoprecipitation					
	RhopH2	CLAG3	3D7‐1	0532300	0301700	3D7‐2
0113700†	0	0	0	12	13	0
0501100†	0	0	0	10	10	0
HSP70x†	23	37	21	53	77	13
0801000†	22	1	0	54	24	5
0532300	3	0	0	26	2	2
0301700	2	0	0	15	22	4
0702500	28	0	0	25	17	1

*Note:* (a) * proteins targeted for reciprocal immunoprecipitation that co‐precipitated RhopH component more strongly than others. (b) Proposed cytoskeletal (CS) complex is indicated with #. (c) The transient J‐dot and Maurer's cleft (JAM) trafficking complex proteins are indicated in the vertical column, with J‐dot protein indicated by †. 3D7‐1 is control used for RhopH2 and CLAG3 assays and 3D7‐2 control for exported proteins targeted. Numbers in each column represent peptides of proteins (protein names displayed vertically) co‐precipitating with proteins targeted for reciprocal immunoprecipitation (protein names displayed horizontally). Extended table can be found in Table S2.

When taken together, peptide count and protein coverage indicated that RESA1, PF3D7_0532300, PF3D7_1201000 and LyMP co‐precipitated all three members of the RhopH complex, whilst the remainder demonstrated association with either RhopH2 only, or no association with any RhopH components (Figure 3a, Tables 1 and S2). To confirm our mass spectrometry analysis, we completed more stringent immunoprecipitation assays using 1% Triton X‐100 to see if the association of these four proteins to RhopH complex was strong and detectable by western blotting (Figure 3b(i)). Western blot analysis was completed for RESA1, PF3D7_0532300, PF3D7_1201000, LyMP, PF3D7_0401800, PF3D7_0501000 and PF3D7_1401200. Blots were probed with antibodies specific for RhopH2, CLAG3 and RhopH3 (see antibody specificity Figure S5) to determine interactions between each of the proteins.

These assays revealed that PF3D7_1401200 showed the strongest association with all RhopH components. RESA1, PF3D7_1201000, PF3D7_0401800 and LyMP also showed interaction with RhopH components but to lesser extent (Figures 3b(i) and S6). PF3D7_0532300 and PF3D7_0501000 showed no interaction with RhopH components when compared to negative control lines, indicating that the PF3D7_0532300/RhopH interaction is lost when using a stronger detergent concentration. None of the five proteins showed strong interaction with RhopH3.

### 3.5 CLAG3 immunoprecipitation confirms the interaction of RESA1, PF3D7_1201000, PF3D7_0401800, LyMP and PF3D7_1401200

CLAG3 was used to further confirm the association of these five exported proteins with RhopH components. CLAG3.2 was tagged with HA*glmS* using standard homologous recombination techniques as described above (Figure S1a,b), targeted for immunoprecipitation and analysed via mass spectrometry. Parasitised RBC were lysed in 1% Triton X‐100 to identify strong associations. Mass spectrometry analysis revealed that RESA1, PF3D7_1201000, PF3D7_0401800, LyMP and PF3D7_1401200 co‐precipitated with CLAG3, whilst PF3D7_0532300 showed no interaction with CLAG3 (Tables 1a and S2). These results further confirm our western blot analysis (Figures 3b(i) and S6).

### 3.6 Comparative immunoprecipitation assays identify two new protein complexes – a cytoskeletal complex and a Maurer's cleft/J‐dot trafficking complex

**FIGURE 4:**
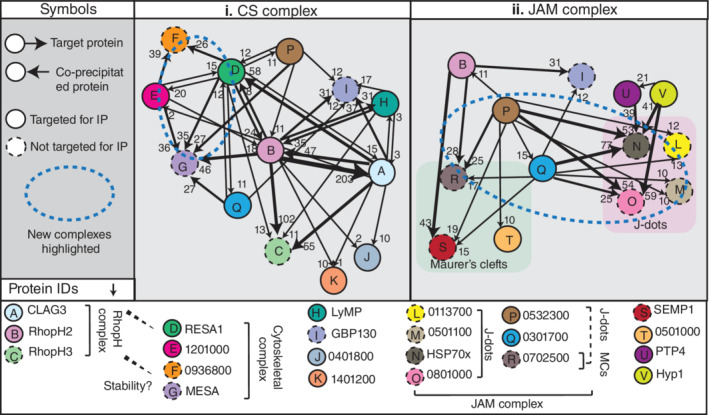
Analysis of immunoprecipitation assays reveal novel protein–protein complexes and trafficking routes within the iRBC. (i) RESA (D) and PF3D7_1201000 (E) are likely in a complex together given the similarity of their interacting partners as determined by mass spectrometry (Tables 1b and S2). Both proteins interact strongly with MESA (G) and PF3D7_0936800 (F). We propose these four proteins are in a cytoskeletal (CS) complex given their interaction with cytoskeletal components and peripheral localisation within the iRBC (dotted blue circle used to highlight the complex). The degree of protein interaction is indicated by thickness of the arrows, and relevant peptide counts co‐precipitated by target protein are represented at the head of the arrow (see symbols in the left panel). (ii) PF3D7_0532300 (P) and PF3D7_0301700 (Q) likely help traffic proteins within the iRBC as both proteins strongly associate with the J‐dot proteins HSP70‐x (N) and PF3D7_0801000 (O). These proteins also interact with the Maurer's cleft resident protein PF3D7_0702500 (R) and these five proteins could therefore form the J‐dot and Maurer's cleft (JAM) complex (dotted blue circle used to highlight the complex). Proteins circled with a dotted line were not targeted for immunoprecipitation here. All peptide numbers for proteins displayed in the figure can be found in Table S2

**FIGURE 5:**
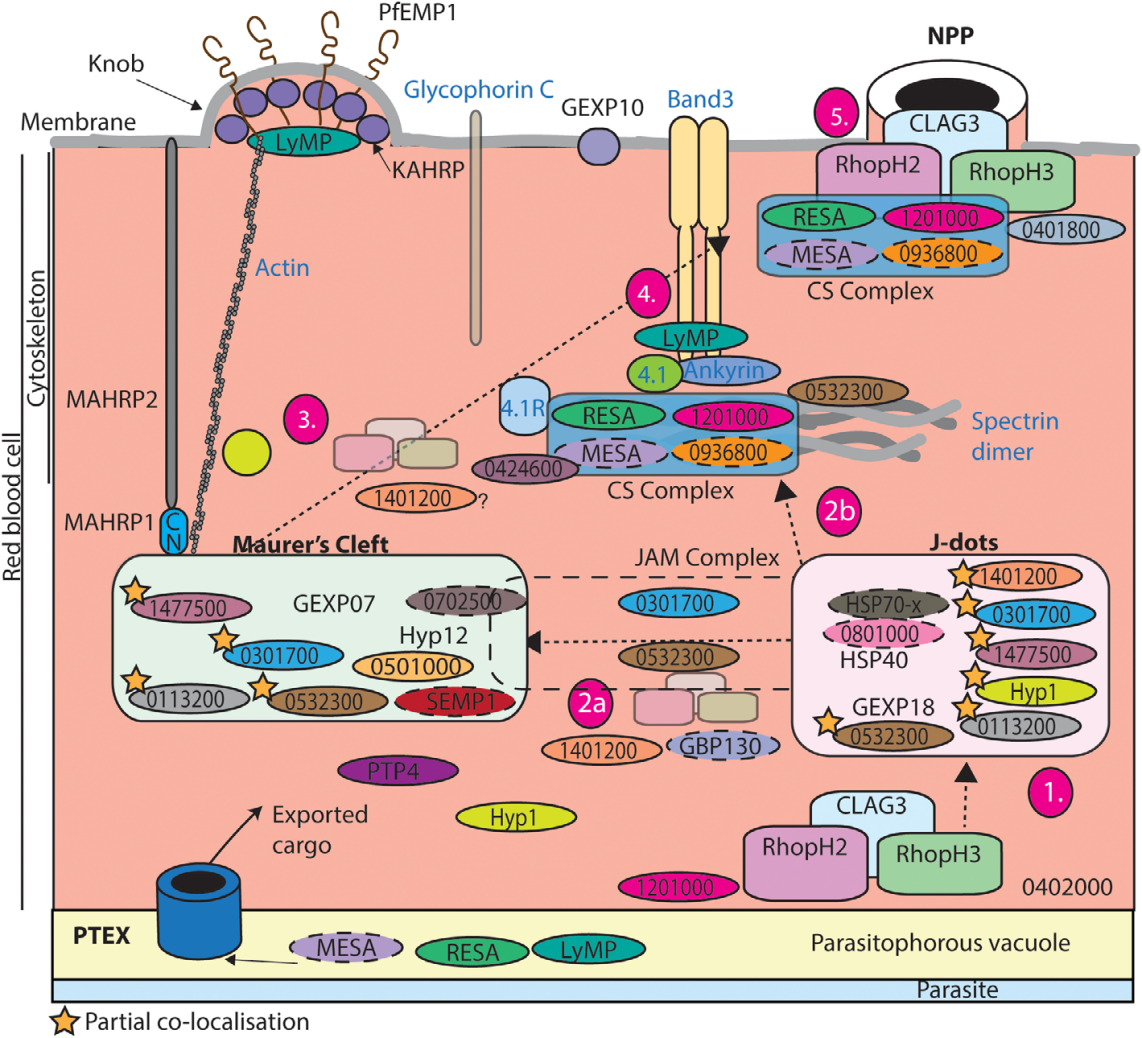
Schematic of protein networks within the iRBC. Schematic figure of protein organisation and potential protein interactions within the iRBC. (1) From immunofluorescence assays it appears that a substantial proportion of the RhopH complex resides at the PV/PVM. This PV localisation could explain its interaction with PF3D7_0402000 (PHISTa), GBP130 and PF3D7_1201000 (PHISTb) proteins. (2) Our data suggests that the JAM complex might help facilitate transport of proteins from J‐dots to Maurer's clefts (MC). J‐dot proteins strongly interacted with the RhopH components but only weak interaction was observed for MC residential proteins. It is therefore unknown as to whether the RhopH proteins are trafficked (2a) through J‐dots to the MC via the JAM complex or (2b) directly from J‐dots to the cytoskeleton/surface. (3) RhopH2 and CLAG3 immunoprecipitation assays show that both components are interacting with PF3D7_1401200, a protein localised to the cytoplasm of the iRBC and could be interacting with the complex due to a trafficking role. (4) Immunoprecipitation assays suggest that RESA1, PF3D7_1201000, MESA and PF3D7_0936800 form a cytoskeletal (CS) complex. (5) The RhopH components could be stabilised at the iRBC membrane by the CS complex and PF3D7_0401800, however, none of the proteins appear to be individually important for the correct functioning of the NPP channels. Parasite proteins are written in black text and host cell proteins in blue text

Reciprocal immunoprecipitation data clarified certain protein–protein interactions occurring within the iRBC cytoplasm and the potential trafficking route of the RhopH complex. The mass spectrometry analysis strongly suggests that RESA1 and PF3D7_1201000 are in a complex together as they show almost identical protein–protein interactions (Figure 4(i), Tables 1b and S2). Both proteins interact strongly with MESA and PF3D7_0936800 and could therefore be in a cytoskeletal (CS) complex given their localisation within the RBC (Figures 4(i), 5, Tables 1b and S2).

Both RhopH2 (Counihan et al., 2017) and CLAG3 co‐immunoprecipitations indicate that GBP130 strongly interacts with both components (Figure 4(i), Table S2). To confirm this, we utilised an antibody against GBP130 for co‐immunoprecipitation assays of magnet‐ purified wild‐type 3D7 parasites. Western blot analysis revealed that GBP130 was only able to co‐precipitate RhopH3 and not the remainder of the RhopH components as would be expected from the mass spectrometry analysis (Figure 3b(ii)). GBP130 might be co‐precipitating indirectly with RhopH2 and CLAG3 due to their association with RhopH3, which is detectable by mass spectrometry but not by western blotting or this could be due to different antibodies used.

Both PF3D7_0532300 and PF3D7_0301700 are likely to serve a trafficking role within the iRBC as both proteins strongly interact with most of the exported proteins on the list, especially the J‐dot chaperones HSP70‐x and PF3D7_0801000. Both proteins also strongly associate with the MC resident protein PF3D7_0702500 and these five proteins therefore could potentially form a J‐dot and MC (JAM) transient complex involved in protein trafficking (Figure 4(ii), Tables 1c and S2). We also note that PF3D7_0532300 strongly co‐precipitated the MC protein REX1 and the major virulence protein PfEMP1 as well as the RBC protein myosin whilst the rest of the proteins showed little or no association with these proteins (Table S2).

## 4 DISCUSSION

Here, we confirm previous observations for the localisation of: RESA1, PF3D7_0401800, PF3D7_0424600, PF3D7_1201000, LyMP, PF3D7_0301700 (Figures 1 and 5) (Davies et al., 2016; Moreira et al., 2016; Proellocks et al., 2014; Schulze et al., 2015; Tarr et al., 2014; Tiburcio et al., 2015). Furthermore, we have also been able to confirm the prediction studies suggesting that PF3D7_0532300, PTP4, PF3D7_1477500, Hyp1, PF3D7_0113200, PF3D7_0501000 and PF3D7_1401200 are exported into the host cell cytoplasm, and demonstrate that they have diffuse or punctate localisation. One protein, PF3D7_0501000, showed complete co‐localisation with REX1 and therefore is an MC resident protein, confirming previous co‐precipitation data (Figures 1 and 5) (McHugh et al., 2020).

Knockdown of the 13 proteins had no effect on sorbitol uptake through the NPPs indicating they are not essential for NPP activity at the knockdown levels achieved in this study (Figure 2a,d). We further used growth assays to assess parasite proliferation during protein knockdown over three consecutive life cycles and determined that PF3D7_1401200 was the only protein with a growth phenotype when compared to wild‐type parasites (Figure 2e). Consistently, PF3D7_1401200 is also the only protein out of the 13 proteins shown to be essential through *piggyBac* transposon mutagenesis screen (Zhang et al., 2018).

The 13 proteins were targeted for reciprocal immunoprecipitation to confirm their interaction with RhopH components. We also targeted CLAG3, a RhopH component, for immunoprecipitation analysis to strengthen our findings. When combining all our immunoprecipitation data, five proteins were confirmed as bona fide RhopH binders: RESA1, PF3D7_1201000, PF3D7_0401800, LyMP and PF3D7_1401200 (Figures 3 and 5). PF3D7_0532300 only interacted with RhopH components under weaker detergent conditions indicating this association is likely weak, transient or indirect (Figure 3). RESA1, PF3D7_1201000, PF3D7_0401800 and LyMP all localise to the iRBC surface and have been shown previously to interact with cytoskeletal components (Figure 5) (Da Silva et al., 1994; Foley et al., 1991; Pei et al., 2007; Proellocks et al., 2014; Silva et al., 2005; Tarr et al., 2014). Only RESA1 and PF3D7_1201000 showed stronger association to cytoskeletal components compared to that of the other exported proteins targeted here and therefore might be associating with RhopH components due to their localisation and not direct association (Table 1b). Cytoskeletal interaction has not been directly shown for PF3D7_1201000 but our mass spectrometry analysis shows that PF3D7_1201000 is strongly associating with spectrin, ankyrin and band 3 (Table 1b). Interestingly, PF3D7_1401200 showed the strongest association with the RhopH components, but is localised within parasite structures that partially co‐localise with MCs and J‐dots (Figures 1a and S2) in the iRBC and is not found at the surface like the remainder of the five RhopH interacting proteins. Knockdown of PF3D7_1401200 resulted in a growth effect in the third cycle of GlcN treatment, where parasites displayed a delayed growth phenotype relative to 3D7 wild‐type parasites (Figure S7a,b). We therefore tested sorbitol uptake after two cycles of GlcN treatment, as changes to NPP activity are observed for RhopH2 knockdown in the cycle prior to any growth effect observed (Counihan et al., 2017). We observed no changes in the rate of iRBC lysis for PF3D7_1401200 compared to 3D7 control line (Figure S7c). We were unable to study the third cycle of treatment as the parasites were younger compared to those not treated with GlcN and would therefore have less active NPPs regardless of PF3D7_1401200 association with the NPPs. Due to this, we did not further characterise the functional association of PF3D7_1401200 with RhopH components.

Mass spectrometry analysis showed that both RESA1 and PF3D7_1201000 strongly associate with MESA and PF3D7_0936800, which are known to interact with cytoskeletal components (Table 1b) (Lustigman et al., 1990; Tarr et al., 2014). We therefore propose that these proteins likely form a cytoskeletal (CS) complex, which serves to stabilise the RhopH complex at the iRBC cytoskeleton (Figures 4(i) and 5). Interestingly, PF3D7_1201000 shows a dual localisation, both at the PVM and iRBC surface, much like the RhopH components (Figures 1a and S5c). We can therefore not exclude the possibility that PF3D7_1201000 also interacts with the RhopH complex at the PVM (Bannister et al., 1986; Counihan et al., 2017; Ito et al., 2017; Vincensini et al., 2008).

PF3D7_0532300 co‐precipitated almost all of the previously identified RhopH2 binders and strongly interacted with the J‐dot proteins HSP70‐x, PF3D7_0801000 and HSP40s (PF3D7_0113700, PF3D7_0501100) (Tables 1c and S2). PF3D7_0532300 also showed partial co‐localisation with the J‐dot protein PF3D7_0801000 by microscopy as well as the MC protein REX1 (Figures 1a and S2). PF3D7_0532300 could form a transient complex with the MC resident proteins PF3D7_0301700, PF3D7_0702500 and J‐dot proteins HSP70‐x and PF3D7_0801000 (Figures 4(ii) and 5). This complex, referred to here as the JAM complex, could serve as a trafficking hub from J‐dots to MCs, as it has been previously suggested that J‐dot structures serve as a bridge for exported proteins to reach the MCs (Kulzer et al., 2010; Petersen et al., 2016). We hypothesise that the RhopH complex could take the route from J‐dots to MCs to access the iRBC surface given the interaction we observed, although formal investigation of this will be required (Counihan et al., 2017; Sam‐Yellowe et al., 2001; Vincensini et al., 2005).

In conclusion, we could not find evidence that the 13 proteins are directly involved in NPP function. We have however, successfully provided a clearer picture of the protein–protein interactions these proteins might be involved in within the iRBC (Figures 4 and 5). As some of these proteins have not been previously tagged and studied, this also adds a new dimension to the current literature and insights into potential protein complexes and networks for future studies. This study also strengthens the link between the two trafficking structures, J‐dots and MCs, and the RhopH complex suggesting it takes this route to the iRBC membrane.

## 5 EXPERIMENTAL PROCEDURES

### 5.1 Plasmid constructs

Roughly 800–1,000 base pairs upstream of the stop codon of each protein was amplified from *P. falciparum* genomic DNA (gDNA) (primer sequences listed in Table S3). This recombination flank was appended with a HA‐tag fused in frame with a T2A skip peptide and a neomycin resistance gene (Birnbaum et al., 2017). This was followed by a *glmS* riboswitch inserted at the heterologous 3′ untranslated region (UTR) of the gene (Prommana et al., 2013) (Figure S1a). Constructs used for standard recombination lacked the T2A skip peptide and neomycin resistance gene. Gene constructs were inserted into a plasmid containing human dihydrofolate reductase (hDHFR), which confers resistance to WR99210. The previously published pPTEX150‐HAglmS plasmid was cut with PstI and BglII to remove the PTEX150 sequence, and recombination flanks were ligated into the plasmid (de Koning‐Ward et al., 2009). These constructs were then transfected into 3D7 wild‐type parasites and placed under selection. When parasite lines were established, gDNA was extracted using DNeasy kit (Qiagen). Correct integration was confirmed by PCR, and correct size of protein by western blotting (Figure S1b–d detailed methods can be found in supplementary materials).

### 5.2 Parasite culturing

Transgenic parasite lines were generated using standard trophozoite transfection as previously described (Fidock & Wellems, 1997). Transgenic parasites were selected for by addition of 2.5 nM WR99210, 5 μg/ml blasticidin S (Sigma Aldrich) and/or 400 μg/ml G418 (geniticin, Sigma Aldrich) and maintained in culture as previously described (Birnbaum et al., 2017, Trager & Jensen, 1976).

### 5.3 Indirect immunofluorescence assays

Infected RBCs were either dropped onto poly‐l‐lysine (Sigma Aldrich) coated coverslips and subsequently fixed in 4% paraformaldehyde/0.0075% glutaraldehyde for 20 min prior to quenching in 0.1 M Glycine/0.1% Triton X‐100, or smeared onto glass slides and fixed in ice‐cold 90% acetone/10% methanol for 2 min. Cells were subsequently blocked in 3% BSA/PBS for 1 hr prior to probing with primary antibodies overnight. Unbound antibodies were removed by extensive washing in 0.02% TX100/PBS prior to addition of secondary antibodies (AlexaFluor) and incubated for 1 hr (antibodies listed in Table S4). Washes were completed as before. Coverslips were mounted on slides with Vectorshield/DAPI. Images were obtained using Zeiss Axio Observer Z1 inverted widefield microscope or a Nikon Eclipse Ti2 microscope and processed using Fiji software. For super resolution images, a Nikon N‐SIM microscope was used (extended protocol can be found in supplemented materials).

### 5.4 Conditional knockdown growth assays

Parasites were treated with varying concentrations of GlcN (0, 0.5, 2.5 mM) at trophozoite stage and adjusted to 1% haematocrit/0.3% parasitemia and plated in a 96‐well plate in 100 μL triplicates. Samples were taken each cycle at trophozoite stage and stored at −80°C until all samples were collected. Parasite growth was estimated by measuring lactate dehydrogenase activity as previously described (Makler et al., 1993; Persson et al., 2006). Experiments were repeated on three independent occasions and completed in technical triplicates. Statistical analysis was performed on the third cycle of treatment for 2.5 mM GlcN only using an unpaired *t‐*test (with Welch correction), where growth was compared to 3D7 wild‐type parasites. Giemsa growth assays were prepared the same way, except smears were taken each day and stained in Giemsa stain.

### 5.5 Sorbitol lysis assays

Transgenic parasite lines expressing exported Nluc (Azevedo et al., 2014) were sorbitol synchronised at ring stage, and the following day when the parasites were trophozoites (~32 hpi) they were treated with varying concentrations of GlcN (0, 0.5 and 2.5 mM) and plated in technical triplicates in 96‐well plates at 1% haematocrit/0.5% parasitemia. In the next cycle when trophozoites (~32 hpi), parasites were incubated in sorbitol lysis buffer (280 mM sorbitol, 0.1 mg/ml BSA, 20 mM Na‐HEPES, pH 7.4) containing NanoGlo substrate (1:1000, Promega) and bioluminescence was monitored every 3 min by CLARIOstar BMG plate reader as described previously (Counihan et al., 2017). For sorbitol lysis assays conducted after two cycles of GlcN treatment, parasites were adjusted to 1% parasitemia and 1% haematocrit on the day of the experiment. All experiments were repeated on three independent occasions and done in technical triplicates.

### 5.6 Reciprocal immunoprecipitation assays analysed via mass spectrometry

RBC infected with trophozoite stage parasites were either purified by passing through a magnetic column (exported proteins) or by saponin lysis (CLAG3). Pellets were lysed in 0.25% TX100 (exported proteins) or 1% Triton X‐100 (CLAG3) and immunoprecipitation assays performed using commercially available anti‐HA agarose beads (Sigma Aldrich). Mass spectrometry‐based proteomics analysis was used to investigate protein–protein interactions for each of the immunoprecipitation assays. A detailed protocol of sample preparation and analysis can be found in the supplementary materials.

### 5.7 Reciprocal immunoprecipitation assays analysed via western blotting

For each tagged protein, 30 ml culture at 4% haematocrit was harvested at trophozoite stage by magnet purification. Infected RBC pellets were washed 2× in PBS containing Complete protease cocktail inhibitors (Roche) and subsequently resuspended in 1% Triton X‐100/PBS lysis buffer. Cells were sonicated 2× cycles (30 s on/30 s off, Diagenode) and incubated in lysis buffer for 1 hr at 4°C. Samples were centrifuged at 20000*g* for 15 min at 4°C and supernatant containing solubilised proteins was incubated with anti‐HA agarose beads (Sigma Aldrich) overnight at 4°C. Beads were washed 5× in lysis buffer and eluted in 1× sample buffer (6X stock: 0.3 M Tris–HCl pH 6.8, 60% flycerole, 12 mM EDTA, 12% SDS, 0.05% bromophenol blue). 100 mM DTT was added to each sample and tubes incubated at 80°C for 10 min and electrophoresed on 4–12% Bis‐Tris SDS‐PAGE gels (Invitrogen) in 1X MOPS buffer, prior to transferring to nitrocellulose membrane for western blotting using iBlot 1 (20 V, 9 min, Invitrogen).

## CONFLICT OF INTEREST

The authors declare no conflicts of interest.

## AUTHOR CONTRIBUTION

T.J. carried out and designed this study, which included experimental procedure, data analysis, figures and manuscript writing unless otherwise indicated. The 11 SLI genetic constructs were generated by P.G. and M.G. and transgenic parasite lines established by T.J. PF3D7_0501000 and PF3D7_1401200 transgenic parasite lines and PCR confirmations were done by N.C. RhopH antibodies and confirmation were generated by J.M. B.K. performed SIM microscopy and analysis. P.S. did mass spectrometry analysis using Bio21 facility for the 10 exported proteins and data was interpreted by T.J. M.G. helped with western blot immunoprecipitation assays. H.B, T.dK‐W, B.C. and P.G. provided overall guidance with the study and manuscript editing.

## Supporting information

**Figure S1.** Generation of transgenic parasite lines. (a) Transgenic parasite lines were either generated using the SLI method using the T2A skip peptide and Neomycin resistance (NeoR) gene or using standard techniques. All proteins were appended with HA protein tag for detection and *glmS* riboswitch for conditional knockdown methods. (b,c) PCR confirmation for each tagged protein to confirm correct insertion, primers are listed in Table S3. (d) Western blots were performed to confirm that the targeted genes were expressing the correctly sized HA‐tagged protein. EXP2 was used as a loading control. N indicated NeoR gene and * target protein.
**Figure S2.** Co‐localisation with J‐dot protein 0801000. Indirect immunofluorescence assay was used to determine co‐localisation of proteins located within the iRBC and the J‐dot marker 0801000. HA is target protein. Scale bars = 5 μm.
**Figure S3.** Supplementary data accompanying Figure 2. (a) Western blots were performed to assess level of protein knockdown when treated *glmS*‐tagged trophozoite stage parasites were treated with 2.5 mM glucosamine (GlcN) over one cycle. Samples were run on four different gels, indicated by numbers. HA antibody was used to detect tagged proteins and EXP2 as loading control. (b) Indirect immunofluorescence assays were used to confirm that the Nluc was successfully exported into the iRBC. HA represents the target protein and Nluc represents the exported Nluc.
**Figure S4.** Malstat growth assay, all cycles. Trophozoite stage parasites were treated with different concentrations of GlcN over three consecutive parasite cycles. Data represent three biological replicates completed in three technical replicates. Error bars represent SD. Only 1401200 showed growth defect in the third cycle of treatment for both 0.5 and 2.5 mM GlcN.
**Figure S5.** Confirmation of RhopH antibodies by western blotting and immunofluorescence assays. (a) Shizont stage parasites were prepared for western blotting and probed with RhopH antibodies to confirm correct size and specificity. All three antibodies showed the correct size. Indirect immunofluorescence assays both of (b) schizont stage and (c) trophozoite stage parasites were used to confirm correct localisation of antibodies within the iRBC. Shizont stage staining was done on 3D7 wild‐type parasite whilst trophozoite stage staining was done on RESA1‐HA‐N‐glmS parasite line, where the HA shows the localisation of RESA. For panel (c), two fixation methods were used (i) paraformaldehyde/glutaraldehyde and (ii) acetone/methanol fixation.
**Figure S6.** Densitometry was used to determine level of co‐precipitation of RhopH components. The level of co‐precipitation of RhopH2 and CLAG3 components were determined by measuring densitometry of input and elution. For each immunoprecipitation, elution was adjusted to input and then taken as a fold increase from 3D7 control line. GBP130 immunoprecipitation was adjusted to 3D7 beads only. We did not perform densitometry for RhopH3 as the antibodies displayed smearing on the blot, which interfered with the densitometry analysis.
**Figure S7.** Knocking down PF3D7_1401200 results in delayed parasite transition from ring to trophozoite stage. (a) 3D7 wild‐type parasites and PFD7_1401200‐HA‐N‐glmS parasites were treated with GlcN at trophozoite stage over three consecutive life cycles and Giemsa smears were taken every 24 hr. PF3D7_1401200 parasites exhibited delayed parasite growth in the third cycle of treatment. No difference was seen for the 3D7 control line. (b) Percentage of parasite stages, (ring, trophozoite and schizont) in the third cycle of GlcN treatment for the PF3D7_1401200 parasite line. (c) PF3D7_1401200‐HA‐N‐glmS_Nluc parasites were treated with different concentrations of GlcN at trophozoite stage. After two cycles of treatment, sorbitol lysis sensitivity was measured and no difference was observed between treatments +/− protein knockdown. Data taken from three biological replicates completed in technical triplicate. Error bars represent standard deviation.

**Table S1.** 30 exported proteins found to interact with RhopH2, Counihan et al. (2017), and their localisation within the iRBC.

Table S2.

**Table S3.** Primer sequences used in this study.

**Table S4.** Antibodies used.

**Appendix S1.** Supporting information.

## Data Availability

The data that support the findings of this study are available from the corresponding author upon reasonable request.
