# Supplementary figures and images for: Characterisation of complexes formed by parasite proteins exported into the host cell compartment of Plasmodium falciparum infected red blood cells
Source: Cell Microbiol. 2021 May 3;23(8):e13332. doi: 10.1111/cmi.13332 (PMC8365696; doi:10.1111/cmi.13332)

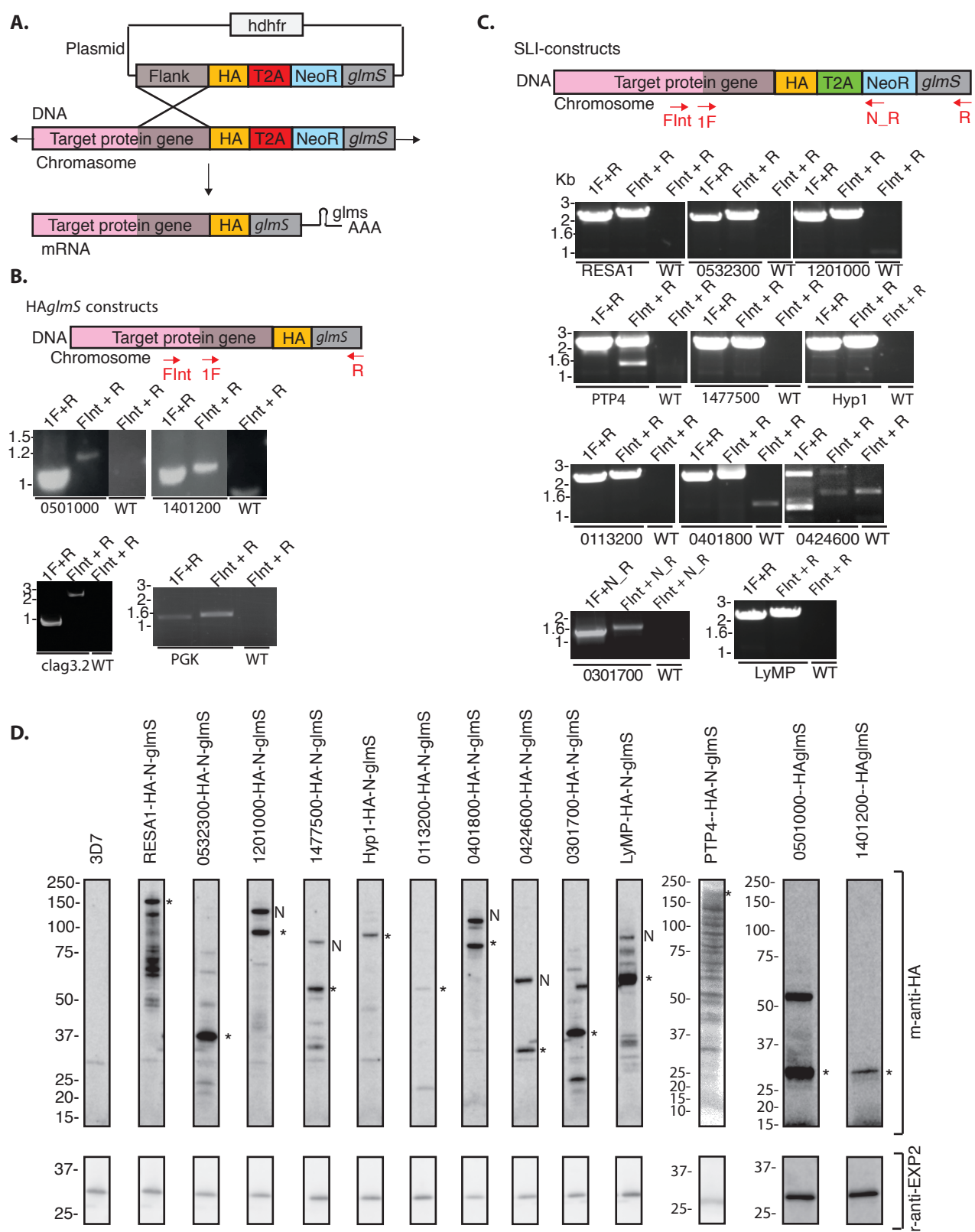

**Figure S1**

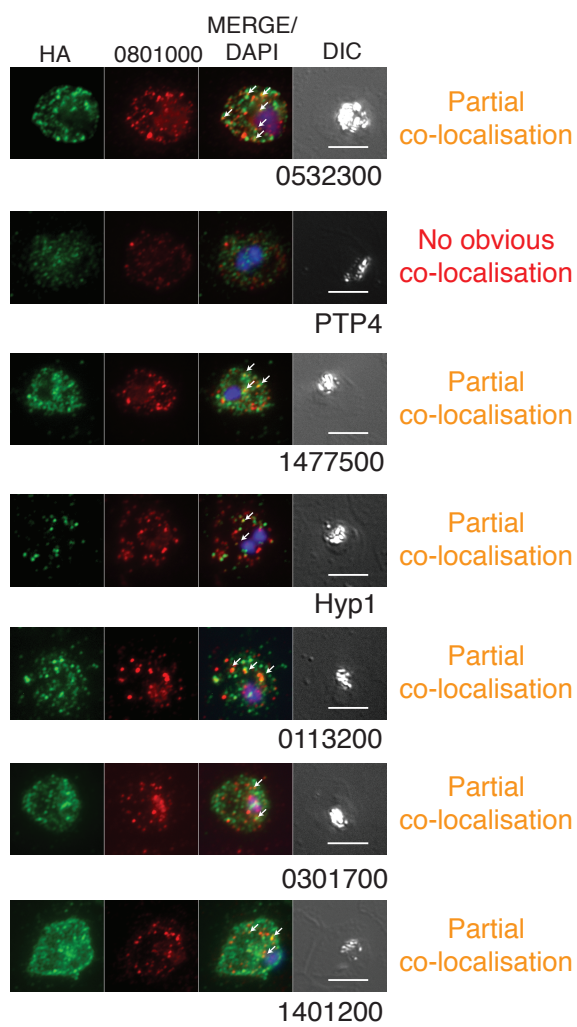

Figure S2

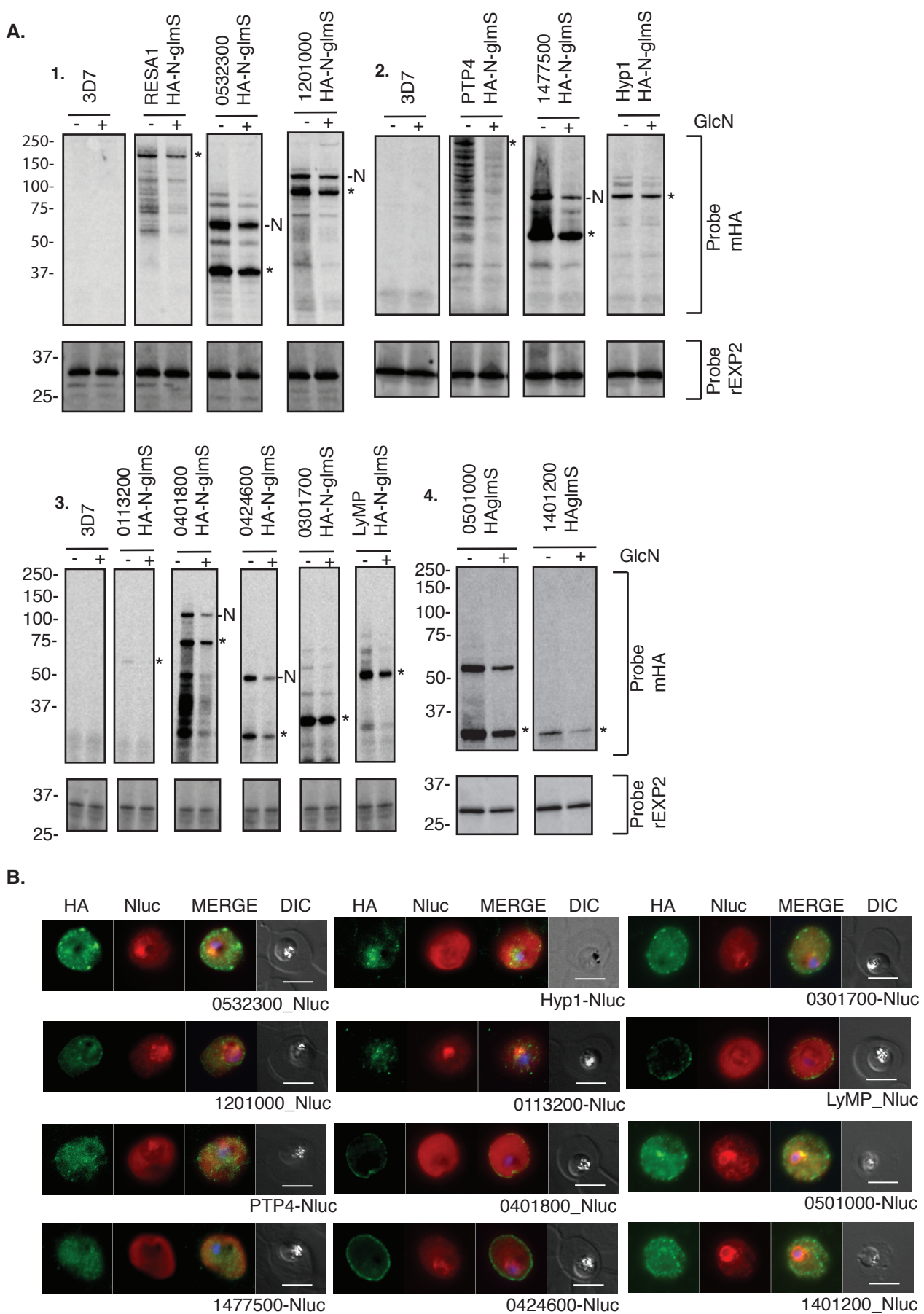

**Figure S3**

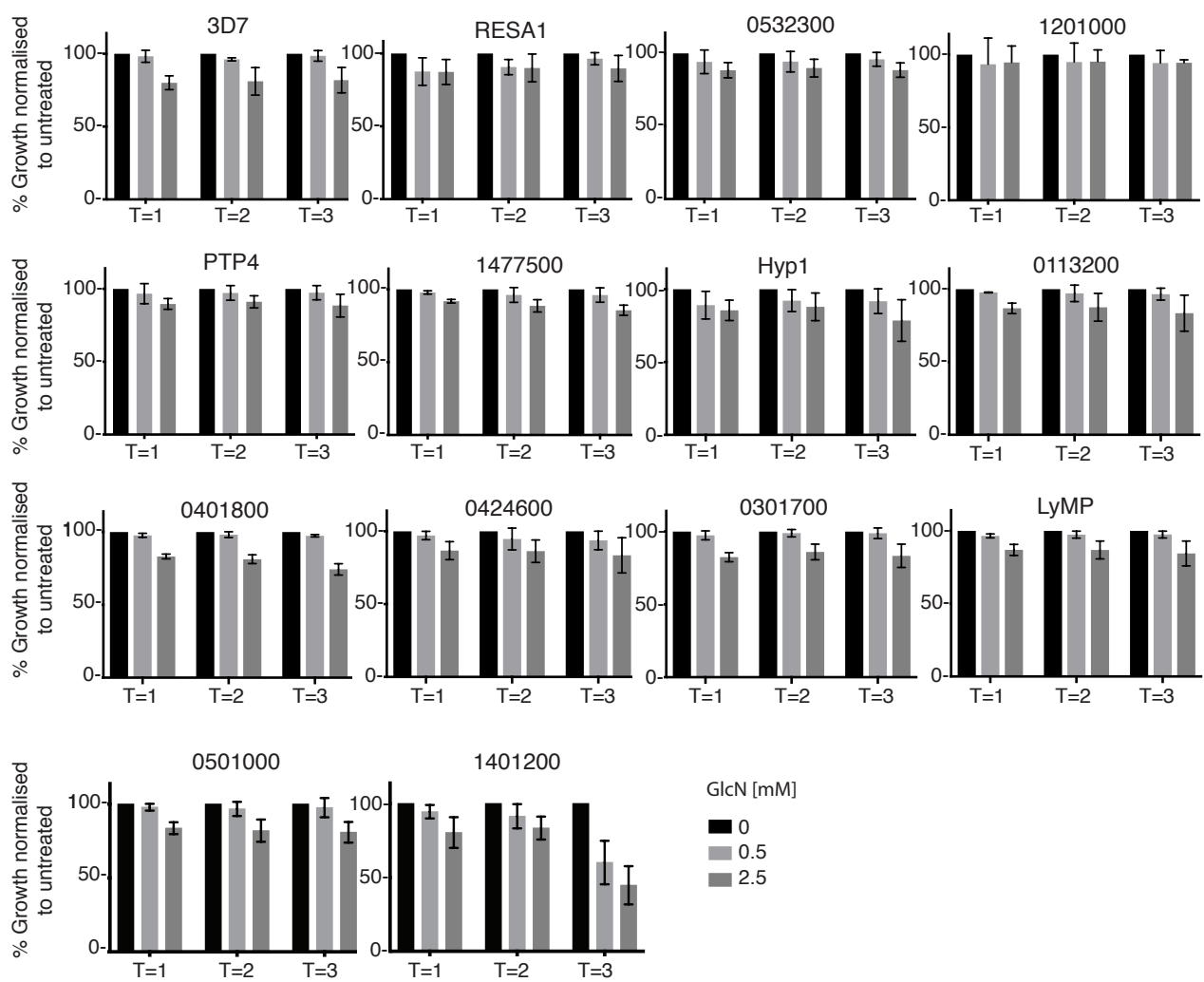

**Figure S4**

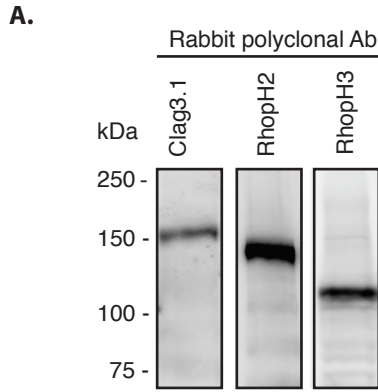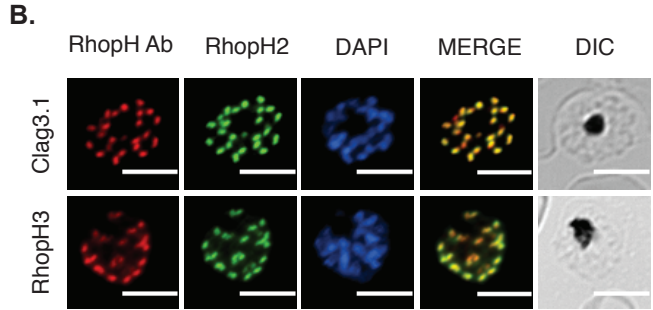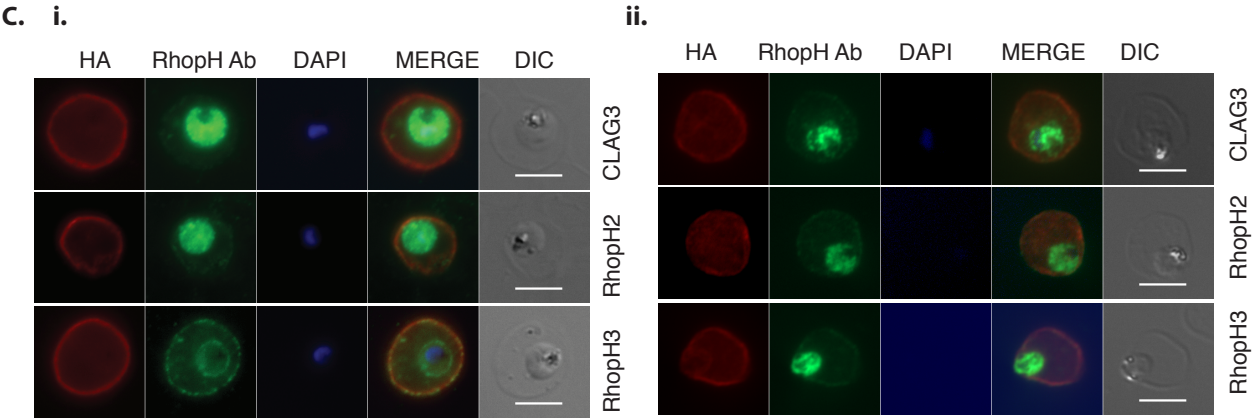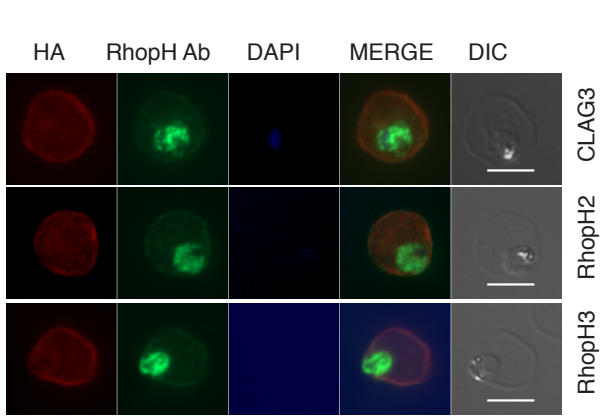

Figure S5

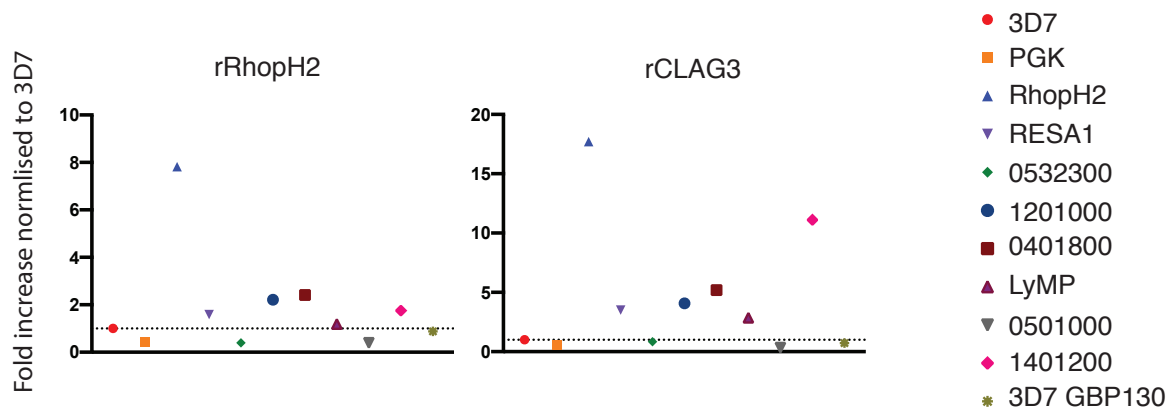

Figure S6

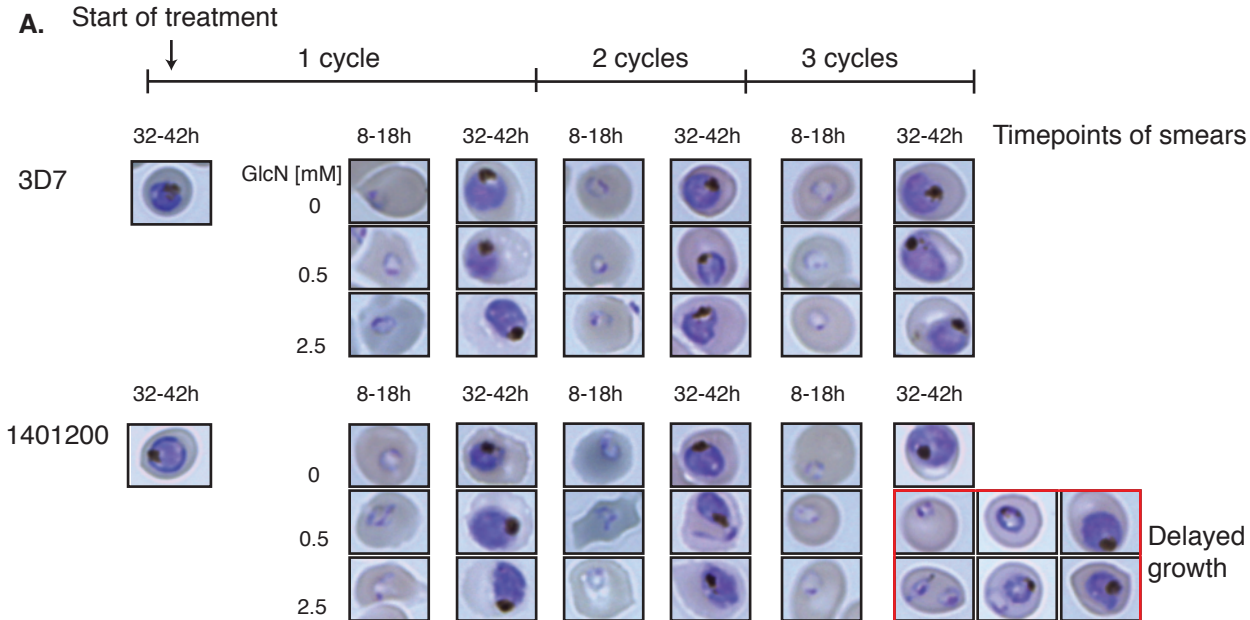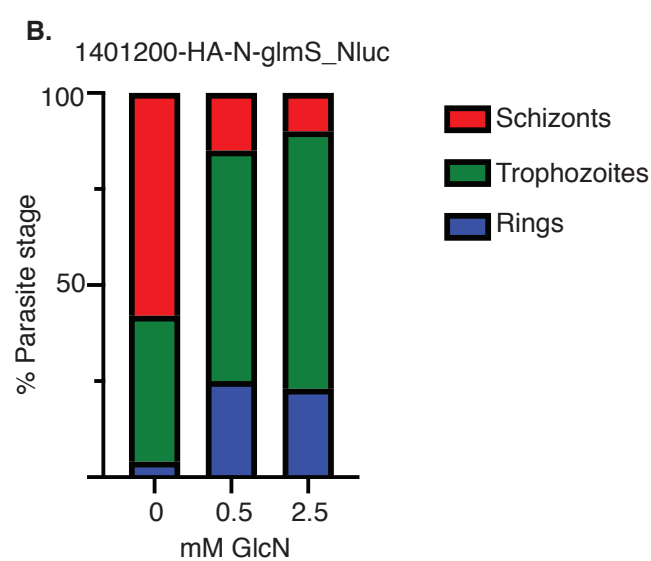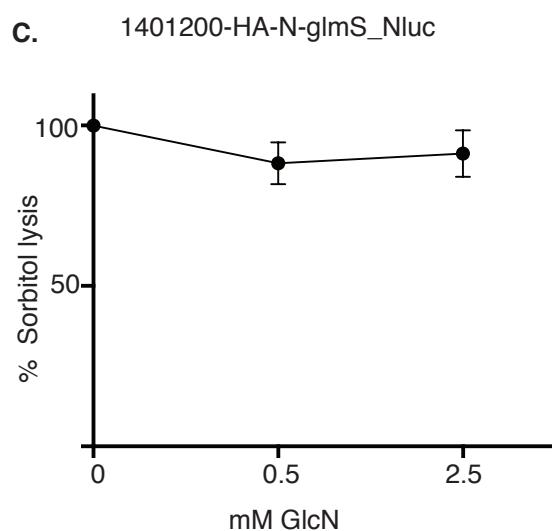

**Figure S7**

Supplement: Supplementary file 1 — Figure S1. Generation of transgenic parasite lines. (a) Transgenic parasite lines were either generated using the SLI method using the T2A skip peptide and Neomycin resistance (NeoR) gene or using standard techniques. All proteins were appended with HA protein tag for detection and glmS riboswitch for conditional knockdown methods. (b,c) PCR confirmation for each tagged protein to confirm correct insertion, primers are listed in Table S3. (d) Western blots were performed to confirm that the targeted genes were expressing the correctly sized HA‐tagged protein. EXP2 was used as a loading control. N indicated NeoR gene and * target protein. Figure S2. Co‐localisation with J‐dot protein 0801000. Indirect immunofluorescence assay was used to determine co‐localisation of proteins located within the iRBC and the J‐dot marker 0801000. HA is target protein. Scale bars = 5 μm. Figure S3. Supplementary data accompanying Figure 2. (a) Western blots were performed to assess level of protein knockdown when treated glmS‐tagged trophozoite stage parasites were treated with 2.5 mM glucosamine (GlcN) over one cycle. Samples were run on four different gels, indicated by numbers. HA antibody was used to detect tagged proteins and EXP2 as loading control. (b) Indirect immunofluorescence assays were used to confirm that the Nluc was successfully exported into the iRBC. HA represents the target protein and Nluc represents the exported Nluc. Figure S4. Malstat growth assay, all cycles. Trophozoite stage parasites were treated with different concentrations of GlcN over three consecutive parasite cycles. Data represent three biological replicates completed in three technical replicates. Error bars represent SD. Only 1401200 showed growth defect in the third cycle of treatment for both 0.5 and 2.5 mM GlcN. Figure S5. Confirmation of RhopH antibodies by western blotting and immunofluorescence assays. (a) Shizont stage parasites were prepared for western blotting and probed with RhopH antibodie [file CMI-23-e13332-s002.pdf]
