# Supplementary material for: Characterisation of complexes formed by parasite proteins exported into the host cell compartment of Plasmodium falciparum infected red blood cells
Source: Cell Microbiol. 2021 May 3;23(8):e13332. doi: 10.1111/cmi.13332 (PMC8365696; doi:10.1111/cmi.13332)
Supplement: Supplementary file 2 — Table S1. 30 exported proteins found to interact with RhopH2, Counihan et al. (2017), and their localisation within the iRBC. [file CMI-23-e13332-s005.docx]

*Table S1: 30 exported proteins found to interact with RhopH2, Counihan et al 2017, and their localisation within the iRBC.*

| **ID** | **Name** | **RhopH2 IP T [R]** | **Essential** | **IFA/proteomic localisation** | **Role** |
| --- | --- | --- | --- | --- | --- |
| **PF3D7_0102200** | RESA1 | 8 [33] | NO (Silva *et al.*, 2005, Maier *et al.*, 2008, Zhang *et al.*, 2018) | RBC, RBC membrane (Tarr *et al.*, 2014, Tiburcio *et al.*, 2015) | Heat shock resistance, iRBC rigidity (Foley *et al.*, 1991, Pei *et al.*, 2007) |
| **PF3D7_0532300** | PHISTb(1) | 3 [0] | NO (Zhang *et al.*, 2018) | RBC and MCs? (Moreira *et al.*, 2016) | Unknown |
| **PF3D7_1201000** | PHISTb(2) | 3 [0] | NO (Maier *et al.*, 2008, Zhang *et al.*, 2018) | PVM, RBC, RBC membrane (Davies *et al.*, 2016) | iRBC rigidity (Maier *et al.*, 2008, Davies *et al.*, 2016) |
| **PF3D7_0730900** | PTP4 | 7 [0] | NO (Maier *et al.*, 2008, Zhang *et al.*, 2018) | RBC, MCs? | PfEMP1 transporter, cytoadherence (Maier *et al.*, 2008) |
| **PF3D7_1477500** | PHISTb(3) | 4 [0] | NO (Zhang *et al.*, 2018) | RBC, MCs? | Unknown |
| **PF3D7_0301600** | Hyp1 | 3 [0] | NO (Zhang *et al.*, 2018) | RBC, MCs? | Unknown |
| **PF3D7_0113200** | - | 3 [0] | NO (Maier *et al.*, 2008, Zhang *et al.*, 2018) | RBC, MCs? | Unknown |
| **PF3D7_0401800** | PHISTb(4) | 2 [1] | YES (Maier *et al.*, 2008), NO (Zhang *et al.*, 2018) | RBC membrane, RBC (Tarr *et al.*, 2014) | Unknown |
| **PF3D7_0424600** | PHISTb(5) | 2 [0] | NO (Maier *et al.*, 2008, Zhang *et al.*, 2018) | RBC membrane, RBC (Tarr *et al.*, 2014) | Knob formation (Maier *et al.*, 2008) |
| **PF3D7_0301700** | - | 2 [0] | NO *(Zhang et al., 2018)* | RBC, MCs (Schulze *et al.*, 2015) | Unknown |
| **PF3D7_0532400** | LyMP | 31 [3] | NO *(Zhang et al., 2018)* | RBC membrane, RBC, transiently with MCs? (Proellocks *et al.*, 2014) | Cytoadherence (Proellocks *et al.*, 2014) |
| **PF3D7_1401200** | - | 10 [0] | YES (Zhang *et al.*, 2018) | Unknown | Unknown |
| **PF3D7_0501000** | - | 7 [0] | NO (Maier *et al.*, 2008, Zhang *et al.*, 2018) | MCs? (McHugh *et al.*, 2020) | Unknown |
| **PF3D7_0402000** | PHISTa | 31 [0] | YES (Zhang *et al.*, 2018), NO (Maier *et al.*, 2008) | PVM (Parish *et al.*, 2013) | Interacts with a sub-population of 4.1R and helps maintain the stability of the PVM? (Parish *et al.*, 2013) |
| **PF3D7_0500800** | MESA | 46 [3] | NO *(Zhang et al., 2018)* | RBC membrane/cytoskeleton (Magowan *et al.*, 1995, Waller *et al.*, 2003) | Binds to protein 4.1 and ankyrin, host cell membrane protein, potential role in cytoskeleton stability (Coppel *et al.*, 1988, Kilili *et al.*, 2019) |
| **PF3D7_0702500** | - | 28 [0] | NO *(Zhang et al., 2018)* | RBC and MCs (Heiber *et al.*, 2013) | Unknown |
| **PF3D7_0831400** | Hyp12 | 26 [0] | NO *(Zhang et al., 2018)* | Unknown | Unknown |
| **PF3D7_0831700** | HSP70-X | 23 [13] | NO (Charnaud *et al.*, 2017, Cobb *et al.*, 2017, Zhang *et al.*, 2018) | PVM, J-dots, partial MCs (Kulzer *et al.*, 2012) | PfEMP1 trafficking, cytoadherence (Charnaud *et al.*, 2017, Day *et al.*, 2019). Important for parasite tolerance to heat shock (Day *et al.*, 2019). |
| **PF3D7_0801000** | PHISTc | 22 [4] | NO *(Zhang et al., 2018)* | J-dots (Zhang *et al.*, 2017) | In a complex with HSP70-X, potential role in cytoadherence (Zhang *et al.*, 2017) |
| **PF3D7_1353100** | - | 18 [2] | NO (Maier *et al.*, 2008, Zhang *et al.*, 2018) | Unknown | Unknown |
| **PF3D7_0702400** | SEMP1 | 43 [11] | NO *(Dietz et al., 2014, Zhang et al., 2018)* | MCs, RBC membrane (Dietz *et al.*, 2014) | No phenotypical changes when knocked out, suggested to be involved in modifications of RBC cytoskeleton (Dietz *et al.*, 2014) |
| **PF3D7_1016400** | FIKK10.1 | 8 [0] | NO (Zhang *et al.*, 2018), YES (Siddiqui *et al.*, 2020) | Unknown | Unknown |
| **PF3D7_1301700** | GEXP07 | 7 [1] | NO (Zhang *et al.*, 2018, McHugh *et al.*, 2020)  YES (Maier *et al.*, 2008) | MCs, External RBC membrane (Sleebs *et al.*, 2014, Hermand *et al.*, 2016) | Co-localises with SBP1(Sleebs *et al.*, 2014). Acts as a ligand to CX3CL1 and contributes to cytoadherence (Hermand *et al.*, 2016). Important for Maurer’s clefts, knobs and PfEMP1 display (McHugh *et al.*, 2020) |
| **PF3D7_1016300** | GBP130 | 31 [1] | NO (Maier *et al.*, 2008, Zhang *et al.*, 2018) | PVM and RBC vesicles (MCs?) (Perkins, 1988) | iRBC rigidity and cytoadherence (Maier *et al.*, 2008) |
| **PF3D7_0936800** | PHISTc | 6 [0] | NO (Zhang *et al.*, 2018)  YES (Maier *et al.*, 2008) | RBC and RBC periphery (Oberli *et al.*, 2014, Schulze *et al.*, 2015) | Binds to the cytoplasmic tail of PfEMP1, co-migrates to knobs (Oberli *et al.*, 2014) |
| **PF3D7_1039000** | FIKK10.2 | 6 [0] | NO (Zhang *et al.*, 2018), YES (Siddiqui *et al.*, 2020) | Unknown | Unknown |
| **PF3D7_0113900** | GEXP10/Hyp8 | 4 [0] | YES (Maier *et al.*, 2008, Zhang *et al.*, 2018, McHugh *et al.*, 2020) | External RBC membrane (Hermand *et al.*, 2016) | Acts as a ligand to CX3CL1 and contributes to cytoadherence (Hermand *et al.*, 2016) |
| **PF3D7_1001000** | Hyp12 | 3 [0] | N/A | Lumen of MCs (Vincensini *et al* 2005) | Unknown |
| **PF3D7_0731300** | PHISTb | 2 [0] | NO (Maier *et al* 2008),  YES (Zhang *et al.*, 2018) | Transiently associated with MCs or RBC membrane (Vincensini *et al.*, 2005) | Unknown |
| **PF3D7_0402400** | GEXP18 | 2 [0] | NO *(Zhang et al., 2018)* | J-dots (Zhang *et al.*, 2017) | In a complex with HSP70-X (Zhang *et al.*, 2017) |

Charnaud, S.C., Dixon, M.W.A., Nie, C.Q., Chappell, L., Sanders, P.R., Nebl, T.*, et al.* (2017). The exported chaperone Hsp70-x supports virulence functions for Plasmodium falciparum blood stage parasites. *PLoS One* **12,** e0181656.

Cobb, D.W., Florentin, A., Fierro, M.A., Krakowiak, M., Moore, J.M. and Muralidharan, V. (2017). The Exported Chaperone PfHsp70x Is Dispensable for the Plasmodium falciparum Intraerythrocytic Life Cycle. *mSphere* **2**.

Coppel, R.L., Lustigman, S., Murray, L. and Anders, R.F. (1988). MESA is a Plasmodium falciparum phosphoprotein associated with the erythrocyte membrane skeleton. *Mol Biochem Parasitol* **31,** 223-231.

Davies, H.M., Thalassinos, K. and Osborne, A.R. (2016). Expansion of Lysine-rich Repeats in Plasmodium Proteins Generates Novel Localization Sequences That Target the Periphery of the Host Erythrocyte. *J Biol Chem* **291,** 26188-26207.

Day, J., Passecker, A., Beck, H.P. and Vakonakis, I. (2019). The Plasmodium falciparum Hsp70-x chaperone assists the heat stress response of the malaria parasite. *FASEB J* **33,** 14611-14624.

Dietz, O., Rusch, S., Brand, F., Mundwiler-Pachlatko, E., Gaida, A., Voss, T. and Beck, H.P. (2014). Characterization of the small exported Plasmodium falciparum membrane protein SEMP1. *PLoS One* **9,** e103272.

Foley, M., Tilley, L., Sawyer, W.H. and Anders, R.F. (1991). The ring-infected erythrocyte surface antigen of Plasmodium falciparum associates with spectrin in the erythrocyte membrane. *Mol Biochem Parasitol* **46,** 137-147.

Heiber, A., Kruse, F., Pick, C., Gruring, C., Flemming, S., Oberli, A.*, et al.* (2013). Identification of new PNEPs indicates a substantial non-PEXEL exportome and underpins common features in Plasmodium falciparum protein export. *PLoS Pathog* **9,** e1003546.

Hermand, P., Ciceron, L., Pionneau, C., Vaquero, C., Combadiere, C. and Deterre, P. (2016). Plasmodium falciparum proteins involved in cytoadherence of infected erythrocytes to chemokine CX3CL1. *Sci Rep* **6,** 33786.

Kilili, G.K., Shakya, B., Dolan, P.T., Wang, L., Husby, M.L., Stahelin, R.V.*, et al.* (2019). The Plasmodium falciparum MESA erythrocyte cytoskeleton-binding (MEC) motif binds to erythrocyte ankyrin. *Mol Biochem Parasitol* **231,** 111189.

Kulzer, S., Charnaud, S., Dagan, T., Riedel, J., Mandal, P., Pesce, E.R.*, et al.* (2012). Plasmodium falciparum-encoded exported hsp70/hsp40 chaperone/co-chaperone complexes within the host erythrocyte. *Cell Microbiol* **14,** 1784-1795.

Magowan, C., Coppel, R.L., Lau, A.O., Moronne, M.M., Tchernia, G. and Mohandas, N. (1995). Role of the Plasmodium falciparum mature-parasite-infected erythrocyte surface antigen (MESA/PfEMP-2) in malarial infection of erythrocytes. *Blood* **86,** 3196-3204.

Maier, A.G., Rug, M., O'Neill, M.T., Brown, M., Chakravorty, S., Szestak, T.*, et al.* (2008). Exported proteins required for virulence and rigidity of Plasmodium falciparum-infected human erythrocytes. *Cell* **134,** 48-61.

McHugh, E., Carmo, O.M.S., Blanch, A., Looker, O., Liu, B., Tiash, S.*, et al.* (2020). Role of Plasmodium falciparum Protein GEXP07 in Maurer's Cleft Morphology, Knob Architecture, and P. falciparum EMP1 Trafficking. *mBio* **11**.

Moreira, C.K., Naissant, B., Coppi, A., Bennett, B.L., Aime, E., Franke-Fayard, B.*, et al.* (2016). The Plasmodium PHIST and RESA-Like Protein Families of Human and Rodent Malaria Parasites. *PLoS One* **11,** e0152510.

Oberli, A., Slater, L.M., Cutts, E., Brand, F., Mundwiler-Pachlatko, E., Rusch, S.*, et al.* (2014). A Plasmodium falciparum PHIST protein binds the virulence factor PfEMP1 and comigrates to knobs on the host cell surface. *FASEB J* **28,** 4420-4433.

Parish, L.A., Mai, D.W., Jones, M.L., Kitson, E.L. and Rayner, J.C. (2013). A member of the Plasmodium falciparum PHIST family binds to the erythrocyte cytoskeleton component band 4.1. *Malar J* **12,** 160.

Pei, X., Guo, X., Coppel, R., Bhattacharjee, S., Haldar, K., Gratzer, W.*, et al.* (2007). The ring-infected erythrocyte surface antigen (RESA) of Plasmodium falciparum stabilizes spectrin tetramers and suppresses further invasion. *Blood* **110,** 1036-1042.

Perkins, M. (1988). Stage-dependent processing and localization of a Plasmodium falciparum protein of 130,000 molecular weight. *Exp Parasitol* **65,** 61-68.

Proellocks, N.I., Herrmann, S., Buckingham, D.W., Hanssen, E., Hodges, E.K., Elsworth, B.*, et al.* (2014). A lysine-rich membrane-associated PHISTb protein involved in alteration of the cytoadhesive properties of Plasmodium falciparum-infected red blood cells. *FASEB J* **28,** 3103-3113.

Schulze, J., Kwiatkowski, M., Borner, J., Schluter, H., Bruchhaus, I., Burmester, T.*, et al.* (2015). The Plasmodium falciparum exportome contains non-canonical PEXEL/HT proteins. *Mol Microbiol* **97,** 301-314.

Siddiqui, G., Proellochs, N.I. and Cooke, B.M. (2020). Identification of essential exported Plasmodium falciparum protein kinases in malaria-infected red blood cells. *Br J Haematol* **188,** 774-783.

Silva, M.D., Cooke, B.M., Guillotte, M., Buckingham, D.W., Sauzet, J.P., Le Scanf, C.*, et al.* (2005). A role for the Plasmodium falciparum RESA protein in resistance against heat shock demonstrated using gene disruption. *Mol Microbiol* **56,** 990-1003.

Sleebs, B.E., Lopaticki, S., Marapana, D.S., O'Neill, M.T., Rajasekaran, P., Gazdik, M.*, et al.* (2014). Inhibition of Plasmepsin V activity demonstrates its essential role in protein export, PfEMP1 display, and survival of malaria parasites. *PLoS Biol* **12,** e1001897.

Tarr, S.J., Moon, R.W., Hardege, I. and Osborne, A.R. (2014). A conserved domain targets exported PHISTb family proteins to the periphery of Plasmodium infected erythrocytes. *Mol Biochem Parasitol* **196,** 29-40.

Tiburcio, M., Dixon, M.W., Looker, O., Younis, S.Y., Tilley, L. and Alano, P. (2015). Specific expression and export of the Plasmodium falciparum Gametocyte EXported Protein-5 marks the gametocyte ring stage. *Malar J* **14,** 334.

Vincensini, L., Richert, S., Blisnick, T., Van Dorsselaer, A., Leize-Wagner, E., Rabilloud, T. and Braun Breton, C. (2005). Proteomic analysis identifies novel proteins of the Maurer's clefts, a secretory compartment delivering Plasmodium falciparum proteins to the surface of its host cell. *Mol Cell Proteomics* **4,** 582-593.

Waller, K.L., Nunomura, W., An, X., Cooke, B.M., Mohandas, N. and Coppel, R.L. (2003). Mature parasite-infected erythrocyte surface antigen (MESA) of Plasmodium falciparum binds to the 30-kDa domain of protein 4.1 in malaria-infected red blood cells. *Blood* **102,** 1911-1914.

Zhang, M., Wang, C., Otto, T.D., Oberstaller, J., Liao, X., Adapa, S.R.*, et al.* (2018). Uncovering the essential genes of the human malaria parasite Plasmodium falciparum by saturation mutagenesis. *Science* **360**.

Zhang, Q., Ma, C., Oberli, A., Zinz, A., Engels, S. and Przyborski, J.M. (2017). Proteomic analysis of exported chaperone/co-chaperone complexes of P. falciparum reveals an array of complex protein-protein interactions. *Sci Rep* **7,** 42188.
