# Supplementary material for: Characterisation of complexes formed by parasite proteins exported into the host cell compartment of Plasmodium falciparum infected red blood cells
Source: Cell Microbiol. 2021 May 3;23(8):e13332. doi: 10.1111/cmi.13332 (PMC8365696; doi:10.1111/cmi.13332)
Supplement: Supplementary file 4 — Table S3. Primer sequences used in this study. [file CMI-23-e13332-s006.docx]

*Table S3: Primer sequences used in this study*

| PF3D7_0102200 (RESA1) - SLI | |
| --- | --- |
| F | GGGAGATCTAAGAAACTTCAACAGAGAACCCAGGC |
| FInt | ATATTACAACCATGTATAGCTGGTGATTCA |
| R | CCCCTGCAGCTTCATCATATTCTTCATTGTGTTCTTCAAC |
| PF3D7_0730900 (PTP4) - SLI | |
| F | GGGAGATCTCATAAATTAAAATCCGACATTGG |
| FInt | GAAATAGGTTTTATGGATGATGATAATAGA |
| R | CCCCTGCAGCTCTTAACATTTTTCTTGGCATGTG |
| PF3D7_1477500 (PHISTb) - SLI | |
| F | GGGAGATCTCGATTTAATGAAAAATGAAATATGG |
| FInt | ATTGAATATACGGAGTATATGCAAAAAGCA |
| R | CCCCTGCAGCAATGGTACCTTTTCTTAATCTTTTATCTC |
| PF3D7_0301600 (Hyp1) - SLI | |
| F | GGGAGATCTCAAATAAAGGTTGAAACG |
| FInt | ACCGGAATATATGTTCTCACAAGAATGGGA |
| R | CCCCTGCAGCATATATATCATATATATTACTGCC |
| PF3D7_0532300 (PHISTb) - SLI | |
| F | GGGAGATCTGATGACATGTGGACAAGATTC |
| FInt | ATCAAATATGGTTGATCGATTAAAGGTACA |
| R | CCCCTGCAGCTTGATTGTTAGAGACTTCATC |
| PF3D7_1201000 (PHISTb) - SLI | |
| F | GGGAGATCTCAAAGAAAAAAGAAACGGAAACG |
| FInt | TGCATGGAAAGCTCTAACTAACTATGCTCT |
| R | CCCCTGCAGCATCTTCATTGTTGAAATATTTAATTATCTTTTG |
| PF3D7_0113200 - SLI | |
| F | GGGAGATCTAAAAACAATTCCGAAGGAC |
| FInt | TTTACATTACTTCAATAGTAATTTTAGAGA |
| R | CCCCTGCAGCAAGAAATAAAAGACATATTGAACC |
| PF3D7_0401800 (PHISTb) - SLI | |
| F | GGGAGATCTCACCATCAACAGGTTCAACATC |
| FInt | TTCAACAGCATCAACAGCATCAACAGGTTC |
| R | CCCCTGCAGCATCTTGTATCATGACTTTAGATTCTTC |
| PF3D7_0424600 (PHISTb) - SLI | |
| F | GGGAGATCTATGGTTATACCTATGTTGGGTG |
| FInt | TTTCTAACTCCAACGGATTTATGGATAGCA |
| R | CCCCTGCAGCTATATCAATAAAATTTTTACTACGC |
| PF3D7_0301700 - SLI | |
| F | GGGAGATCTATATATGCTTTGTGTATTCC |
| FInt | TGTACAATTTTTATCTGGATATGTCAATGT |
| R | CCCCTGCAGCATTTTTTTTTGATTTTTTATTATTATTGTTG |
| PF3D7_0532400 (LyMP) - SLI | |
| F | GGGAGATCTCATATTATAGATTCTATGAAATATATATGGAATG |
| FInt | GATTTAAAGGAGCACGACTTATGGAATGATCA |
| R | CCCCTGCAGCATTTTTTTTTTTATTTTCTTTTCCAG |
| PF3D7_0501000 | |
| F | TGCAAGATCTATGATGAATAAAAAATCA |
| FInt | TACAAAGCAAGGAAAATAT |
| R | TAAGCTGCAGCTTTATTTGATTCTTGTTC |
| PF3D7_1401200 | |
| F | TCGAGGTACCATGTTCCCTTCTTATATTAGA |
| FInt | TATATTATCCATCAGCATAATT |
| R | TAAGCTGCAGCAAATAAACAGGATATACATC |
| PF3D7_0302200 (CLAG3.2) | |
| F | GCGAGATCTCAACATTTGATTTGCGCTTTTGAT |
| FInt | GTGGAACAAAGTTTTGAAATCAT |
| R | GCGCTGCAGCATGAAATCGTGCGATCATTAATTC |
| PF3D7_0922500 (PGK) | |
| F | AAAGATCTTATTAATATCCCATTGTGGTAGA |
| FInt | ACGAACAGAATTACTGCTACATTACCCACAATTAACCA |
| R | TGCAGCTTTGTTTGAAAGTGCTAATACACCTGGT |
| PF3D7_0302500 (CLAG3.1) recombinant protein primer | |
| MBP-F | ATGGGATCCATGATTGGAAATGATGAACTAC |
| MBP-R | CAACTGCAGTATTAAATGAGGACCTAGATGATT |
| PF3D7_0929400 (RHOPH2) recombinant protein primer | |
| MBP-F | GTTGGATCCAGTGCAGATAGCAATACGAAG |
| MBP-R | CAACTGCAGATCTAAGAATGCTGGTATCCC |
| PF3D7_0905400 (RHOPH3) recombinant protein primer | |
| MBP-F | ATGGGATCCGTGGATGAACCTGAACAATTTTAC |
| MBP-R | CAAACTAGTGTTAGGTATATCATCATGGAATGA |
| Tags | |
| 3X HA | TACCCGTACGACGTCCCGGACTACGCTGGCTATCCCTATGATGT GCCCGATTATGCGTATCCGTACGATGTTCCAGATTATGCC |
| glmS_F | GCTGCAGCTTACCCGTACGA |
| glmS_R | AGATCATGTGATTTCTCTTTGTTCA |
| N_R | CCCAGTCATAGCCGAATAGCCTCTC |
| 2A | GGAAGCGGAGCTACTAACTTCAGCCTGCTGAAGCAGGCTGGAG ACGTGGAGGAGAACCCTGGACCT |
| NeoR | ATGATTGAACAAGATGGATTGCACGCAGGTTCTCCGGCCGCTTG  GGTGGAGAGGCTATTCGGCTATGACTGGGCACAACAGACAATC  GGCTGCTCTGATGCCGCCGTGTTCCGGCTGTCAGCGCAGGGGC  GCCCGGTTCTTTTTGTCAAGACCGACCTGTCCGGTGCCCTGAAT  GAACTACAGGACGAGGCAGCGCGGCTATCGTGGCTGGCCACGA  CGGGCGTTCCTTGCGCAGCTGTGCTCGACGTTGTCACTGAAGCG  GGAAGGGACTGGCTGCTATTGGGCGAAGTGCCGGGGCAGGATC  TCCTGTCATCTCACCTTGCTCCTGCCGAGAAAGTATCCATCATGG  CTGATGCAATGCGGCGGCTGCATACGCTTGATCCGGCTACCTGC  CCATTCGACCACCAAGCGAAACATCGCATCGAGCGAGCACGTAC  TCGGATGGAAGCCGGTCTTGTCGATCAGGATGATCTGGACGAAG  AGCATCAGGGGCTCGCGCCAGCCGAACTGTTCGCCAGGCTCAAG  GCGCGCATGCCCGACGGCGAGGATCTCGTCGTGACCCATGGCGA  TGCCTGCTTGCCGAATATCATGGTGGAAAATGGCCGCTTTTCTGG  ATTCATCGACTGTGGCCGGCTGGGTGTGGCGGACCGCTATCAGG  ACATAGCGTTGGCTACCCGTGATATTGCTGAAGAGCTTGGCGGCG  AATGGGCTGACCGCTTCCTCGTGCTTTACGGTATCGCCGCTCCCG  ATTCGCAGCGCATCGCCTTCTATCGCCTTCTTGACGAGTTCTTCTG  A |
| *glmS* | TAATTATAGCGCCCGAACTAAGCGCCCGGAAAAAGGCTTAGTTGACG  AGGATGGAGGTTATCGAATTTTCGGCGGATGCCTCCCGGCTGAGTGT  GCAGATCACAGCCGTAAGGATTTCTTCAAACCAAGGGGGTGACTCCTT  GAACAAAGAGAAATCACATGATCT |
