# Supplementary material for: Characterisation of complexes formed by parasite proteins exported into the host cell compartment of Plasmodium falciparum infected red blood cells
Source: Cell Microbiol. 2021 May 3;23(8):e13332. doi: 10.1111/cmi.13332 (PMC8365696; doi:10.1111/cmi.13332)
Supplement: Supplementary file 5 — Table S4. Antibodies used. [file CMI-23-e13332-s001.docx]

*Table S4: Antibodies used*

| **Antibody** | **Immunofluorescence** | **Western blotting** | **Supplier** |
| --- | --- | --- | --- |
| Primary antibodies used | | | |
| m HA | 1:1000 | 1:1000 | Sigma |
| r EXP2 | 1:1000 | 1:1000 | WEHI monoclonal facility |
| m REX1 | 1:500 | - | Gifted from Matt Dixon, Bio21 |
| r 0801000 | 1:1000 | - | WEHI monoclonal facility |
| r IgG Nluc | 1:200 | - | In-house |
| r polyclonal RhopH2 | 1:1000 | 1:300/1:1000 | In-house |
| r polyclonal CLAG3 | 1:1000 | 1:300/1:1000 | In-house |
| r polyclonal kill bleed RhopH3 | 1:1000 | 1:200/1:500 | In-house |
| m GBP130 | - | 1:500 | WEHI monoclonal facility |
| Secondary antibodies used | | | |
| m 594 | 1:2000 | - | AlexaFluor |
| m 564 | 1:2000 | - | AlexaFluor |
| r 488 | 1:2000 | - | AlexaFluor |
| r 700 | - | 1:10 000 | Invitrogen |
| m 800 | - | 1:10 000 | Invitrogen |
| r HRP | - | 1:10 000 | Abcam |
| m HRP | - | 1:10 000 | Abcam |

r= rabbit, m= mouse
