# Supplementary material for: Characterisation of complexes formed by parasite proteins exported into the host cell compartment of Plasmodium falciparum infected red blood cells
Source: Cell Microbiol. 2021 May 3;23(8):e13332. doi: 10.1111/cmi.13332 (PMC8365696; doi:10.1111/cmi.13332)
Supplement: Supplementary file 6 — Appendix S1. Supporting information. [file CMI-23-e13332-s003.docx]

**­Supplementary Methods**

*PCR confirmation of plasmid integration*

To confirm correct integration of the HA-Neo-glmS/HAglmS constructs, schizont stage parasite cultures (~3-5% parasitemia) were treated with 0.3% saponin and gDNA extracted (DNeasy, Quiagen). Standard kit protocol was followed, except an additional centrifugation step (17.000g/10 min) was added following incubation with proteinase k to remove hemozoin crystals. To confirm correct plasmid integration, gDNA was subsequently used for PCR with specific primers (Table S3, Fig. S1B, C). 3D7 WT parasites were used as a negative control for the integration.

*Structural illumination microscopy*

Fluorescence images were obtained by using an inverted microscope (TI-E, Tokyo, Japan) equipped with a motorized piezo stage, Perfect Focus System, Nikon Intensilight E, SIM illuminator, and SIM microscope enclosure. Images were collected using a Plan Apo VC 100x 1.4NA oil objective and using 2D-SIM and 3D-SIM imaging modes with diffraction grating 3D 1 Layer for 2D/3D SIM. The HA tagged protein was excited by a 561-nm solid-state laser (100mW) and EXP2 using a 488-nm solid-state laser (100mW). The images were passed through an emission filter (SIM488 470-490, 500-545) and (SIM561 556-566, 570-640); and captured consecutively by an EM-CCD camera IXON DU897 (Andor) giving a 16 µm x 16 µm, where images have a dimension of 1024 x 1024 pixels giving an area of 32.84 µm x 32.84 µm.

Super-resolution images were acquired using a Nikon N-SIM – Motorised Ti-E inverted microscope with Perfect Focus System and SIM illuminator and SIM microscope enclosure. Three available laser lines, Coherent Sapphire 488 nm (100mW), Coherent Sapphire 561 nm (100mW) and Coherent OBIS 640 nm (100mW). Objectives: SR Plan Apo 100x 1.4 oil, Apo TIRF 100x 1.49 oil SR Plan Apo IR 60x WI. Camera: EM-CCD camera iXON DU897 (Andor) giving a 16 µm x 16 µm (with a 2.5x relay lens). N-SIM Dichroic cubes: N-SIM 488, N-SIM 561, and N-SIM 640. Imaging Modes: TIRF-SIM, 2D-SIM, 3D-SIM. Diffraction Gratings: 3D 1 Layer for 2D/3D SIM with 100x/1.49 objective, excitation wavelength 405-640 nm, EX-V-R for 1D to 3D SIM with 60x/1.27 WI objective, excitation wavelength 405-640 nm, and TIRF SIM with 100x/1.49 objective, an excitation wavelength of 488 nm. TIRF 561 with 100x/1.49 objective for TIRF SIM, excitation wavelength 561 nm. Resolution: Lateral (XY) ~ 85-110 nm (dependent on wavelength and optics) and Axial (Z) ~ 200-250 nm (dependent on wavelength and optics), 3D Axial range up to 20 µm. Speed: TIRF and 2D SIM = 0.6 sec/frame (dependant on exposure time) and 3D SIM = 1.0 sec/frame (dependant on exposure time).

*Western blotting*

RBCs infected with trophozoite stage parasites were passed through a MACS magnetic column (Militenyi Biotech) to isolate late stage parasites containing hemozoin crystals. Pellets were then washed in phosphate buffer saline (PBS) containing Complete protease inhibitor cocktail (Roche) and subsequently resuspended in sample buffer (6X stock: 0.3 M Tris-HCl pH 6.8, 60% flycerole, 12 mM EDTA, 12% SDS, 0.05% bromophenol blue) prior to 3 cycles of sonication (30 s on/30 s off, Diagenode). Samples were reduced by addition of 100 mM DTT and subsequent incubation at 80°C for 10 min, prior to electrophoresis on NuPAGE 4-12% Bis-Tris SDS-PAGE gels (Invitrogen) in 1X MOPS buffer at 200V for 1 h. Proteins were transferred to nitrocellulose membrane either via the iBlot dry transfer system (20V, 7 min, Invitrogen) or overnight wet transfer using the XCell II Blot Module and wet transfer buffer (Tris-glycine buffer with 20% methanol). Membranes were blocked for 1 h in 1% casein/PBS and probed overnight with primary antibodies. Membranes were washed 3x in PBS and probed with fluorescent goat-anti-mouse/rabbit IgG secondary antibodies or HRP antibodies. When using HRP antibodies, membrane was subsequently incubated with chemiluminescence substrate (Thermo Fisher) for 5 min. Labelling was visualised using the LiCor Odyssey imager. See Table S4 for all antibodies used.

To confirm specificity of RhopH antibodies, iRBCs containing schizont stage parasites were lysed in 0.05% saponin (w/v) in PBS. Parasite pellets were solubilised and reduced in Laemmli sample buffer containing 100 mM DTT. The lysates were run on 8% SDS-PAGE gels and transferred to nitrocellulose membranes by wet transfer. The membrane was blocked and probed as described above.

*Conditional knockdown growth assays*

Trophozoite stage parasites were treated with varying concentrations of GlcN (0, 0.5, 2.5 mM) and adjusted to 1% haematocrit/0.3% parasitemia and plated on a 96-well plate in 100 µl triplicates. Each cycle at trophozoite stage, samples were taken and stored at -80°C for later analysis, and remainder of culture was diluted 1 in 8 to be monitored for further cycles. Parasite growth was estimated by measuring lactate dehydrogenase activity (Makler *et al.*, 1993b). Nitroblue tetrazolium (2mg/ml), phenazine ethosulfate (0.1 mg/ml) and malstat reagent were mixed in the ratio 1:1:10 to make up malstat mixture. 30 µl of lysed parasite culture was resuspended in 75 µl of malstat mixture and incubated for 30 min in the dark or until colour change occurred [adapted from (Persson *et al.*, 2006)]. Absorbance was measured at 650 nm using the Multiscan Go Microplate Spectrophotometer (Thermo Fisher). Growth was measured from timepoint 0, when assay was set up and normalised to untreated parasites (100% growth). Assay was completed on three independent occasions in technical triplicates. Statistical analysis was performed on the third cycle of treatment for the highest concentration of GlcN using unpaired t test (Welch’s t test).

*Gene cloning and protein expression of RhopH polyclonal antibodies*

The DNA sequence corresponding to amino acid residues 62-285 of Clag3.1, 38-265 of RhopH2, and 64-356 of RhopH3 were amplified from *P. falciparum* 3D7 gDNA. The amplified Clag3.1 and RhopH2 products were ligated into the BamHI and PstI sites of a modified version of pMal-c2x (New England Biolabs) containing a C-terminal 6× His tag. For RhopH3, the amplified product was cloned into the BamHI and SpeI sites of the same vector. Fusion proteins were expressed in *Escherichia coli* strain BL21 (DE3) (Thermo Fisher) by induction with 1 mM isopropyl β-D-thiogalctopyranoside (Sigma Aldrich). Recombinant proteins were extracted from inclusion bodies by washing the pellets 3x with 2M urea/2% TX100 in buffer A (20 mM Tris/HCl pH 8.0, 1 mM PMFS) and then solubilising in 8 mM urea in buffer A overnight at 4º C while mixing. Samples were clarified by centrifugation at 20 000 g/30 min/4ºC, and purified over nickel resin (Sigma Aldrich) using a 5 mm HiTrap Chelating HP column (GE Healthcare). The concentration of purified solubilised proteins was determined by quick start Bradford protein assay (Bio-Rad). Denatured recombinant Clag3.1, RhopH2 and RhopH3 proteins (60 mg) were then refolded in 250 mL buffer containing 3 M urea, 100 mM Tris–HCl pH 8.0, 0.4 M L-arginine monohydrochloride, 20 mM reduced L-glutathione and 2 mM oxidized L-glutathione by incubating at 4º C with continuous mixing for 48 h prior to dialysing against PBS. Protein purity and size was confirmed by SDS-PAGE. The purified proteins were injected into rabbits to produce polyclonal antibodies at the WEHI antibody facility, Australia.

*Reciprocal immunoprecipitation assays by mass spectrometry (sample preparation)*

10 exported proteins

Parasites were grown in 30ml culture at 4% haematocrit and harvested at trophozoite stage by magnet purification (MACS magnetic column, Militenyi Biotech). Infected RBC pellets were washed 2x in PBS containing Complete protease inhibitors (Roche) and stored at -80°C. Pellets were resuspended in 25x pellet volume 0.25% TX100 lysis buffer, sonicated 3 cycles (30 sec on/ 30 sec off, Diagenode) and kept rotating at 4°C for 1 h. Insoluble material was subsequently pelleted by centrifugation at 14 000g for 10 min at 4°C. Parasite lysate was incubated with 60µl 50:50 slurry of anti-HA agarose beads (Sigma Aldrich) and incubated rotating overnight at 4°C. Samples were centrifuged at 1000g for 1 min at 4°C and unbound fraction removed. Beads were resuspended in 500µl lysis buffer and transferred to a micro-bio-spin column (Bio-Rad) and centrifuged at 500g for 30 sec. Column was washed 3x in 500µl lysis buffer and 3x 500µl TEAB (tris(2-carboxyethyl)phosphine). Beads were resuspended in 500µl TEAB, transferred to an eppendorf tube and centrifuged at 500g for 5 min. Supernatant was removed and beads resuspended in 150µl 8M UREA/10mM TCEP in 50 mM TEAB and left shaking at 800 RPM for 45 min at 37°C. Iodacetaminde/50 mM TEAB was added to samples (final concentration 55mM) followed by incubation shaking at 800 RPM for 30 min at RT in the dark. TEAB (25mM) was added to samples, diluting the UREA concentration from 8M to 1M, followed by trypsin/TEAB (2.8µg) and overnight incubation at 800 RPM at 37°C. Samples were centrifuged 17.000g for 5 min and supernatant incubated with TFA (trifluoroacetic acid) prior to loading onto Pierce C-18 reverse phase columns (Thermo Fisher) and centrifuged 2000g for 30 sec. Samples were washed 3x in 500µl 0.1% TFA and eluted in 400µl elution buffer (80% acetonitrile (Sigma, mass spec grade), 0.1% TFA). Samples were concentrated to 20μl by SpeedVacTM (Thermo Scientific). LC-MS/MS was performed using Orbitrap Lumos mass spectrometer (Thermo Scientific) fitted with nanoflow reversed-phase HPLC (Ultimate 3000 RSLC, Dionex). The nano-LC system was equipped with an Acclaim Pepmap nano-trap column and an Acclaim Pepmap RSLC analytical column. 1 μL of the peptide mix was loaded onto the enrichment (trap) column at an isocratic flow of 5 μL/min of 3% acetonitrile containing 0.1% formic acid for 6 min before the enrichment column was switched in-line with the analytical column. The eluents used for the LC were 0.1% v/v formic acid (solvent A) and 100% acetonitrile/0.1% formic acid v/v. The gradient used was 3% B to 20% B for 95 min, 20% B to 40% B in 10 min, 40% B to 80% B in 5 min and maintained at 80% B for the final 5 min before equilibration for 10 min at 3% B prior to the next sample. The mass spectrometer was equipped with a NanoEsi nano-electrospray ion source (Thermo Fisher, USA) for automated MS/MS. High mass accuracy MS data were obtained in a data-dependent acquisition mode with the Orbitrap resolution set at 75,000 and the top-ten multiply charged species selected for fragmentation by HCD (single-charged and double-charged species were ignored). The ion threshold was set to 15,000 counts for MS/MS. The CE voltage was set to 27. The resolution was set to 120000 at MS1 with lock mass of 445.12003 with HCD Fragmentation and MS2 scan in ion trap. Top three second method was used to select species for fragmentation. Singly charged species were ignored and an ion threshold triggering at 1e4 was employed. CE voltage was set to 1.9kv.

CLAG3.2-HA*glmS* immunoprecipitation

Trophozoite stage *P. falciparum* iRBCs were lysed in 0.05 % saponin (w/v) in PBS and solubilised with 1% (v/v) TX100 in PBS containing Complete protease inhibitors (Roche) for 30 min on ice. Proteins were liberated by sequential freeze-thaw cycles in liquid nitrogen followed by centrifugation at 21 000 g for 10 min at 4°C. Protein concentration was determined by quick start Bradford protein assay (Bio-Rad). Supernatants were incubated with anti-HA agarose beads (Sigma Aldrich) for 8 h at 4°C. Beads were washed 4x with 0.5 % TX100/PBS containing Complete protease inhibitors (Roche) and eluted in 1X sample buffer (6X stock: 0.3 M Tris-HCl pH 6.8, 60% flycerole, 12 mM EDTA, 12% SDS, 0.05% bromophenol blue) containing 100 mM DTT before being electrophoresed by standard SDS-PAGE with Nu-PAGE 4-12 % Bis-Tris polyacrylamide gels (Life Technologies). Following electrophoresis, proteins were stained overnight in Silver stain (Pierce) and de-stained according to manufacturer’s instructions. Bands of interest were excised and stored at -20°C for later analysis. The stored samples were later subjected to in gel-processing by reduction with 10 mM DTT for 30 minutes, alkylation for 30 minutes with 50 mM iodoacetic acid and digestion with 375 ng tryspin (Promega) for 16 hours at 37°C. These samples underwent acidification in 0.1% (v/v) formic acid and were concentrated to approximately 10 µL by centrifugal lyophilization using a SpeedVac AES 1010 (Savant). The extracted protein samples were then introduced by injection and fractionated by nanoflow reversed-phase liquid chromatography on a nano-UHPLC system (Easy n-LC II, Thermo Fisher, USA). This HPLC system was coupled online to an LTQ-Orbitrap mass spectrometer equipped with nanoelectrospray ion source (Thermo Fisher, USA) for automated mass spectrometry.

*Mass spectrometry analysis*

MS data from LC-MS/MS were searched against a non-redundant protein database (Swiss Prot) comprising sequences from the latest version of LudwigNR (Human, Bovine, P. falciparum species), as well as their reverse sequence. Peak lists for each nano-LC-MS/MS run were extracted from Msile2.1 (Proteomics analyser tool from Bio21) which were then used to search MASCOT v2.2.04 algorithm (Matrix Science, UK) provided by the Australian Proteomics Computational Facility using the following parameters: minimum mass 400; maximum mass 5000; grouping tolerance 0.01 Da; intermediate scans 1; minimum group count 1; 10 peaks minimum and total ion current of 100. The search parameters enlisted were carboxymethylation of cysteine as a fixed modification (+58 Da, for gel samples only), with variable modifications set for NH2-terminal acetylation (+42 Da) and oxidation of methionine (+16 Da). A precursor mass tolerance of 20 ppm, #13C defined as 1, fragment ion mass tolerance of ± 0.8 Da, and an allowance for up to three missed cleavages for tryptic searches were also set up.
